# Plant families exhibit unique geographic trends in C_4_ richness and cover in Australia

**DOI:** 10.1371/journal.pone.0271603

**Published:** 2022-08-22

**Authors:** Samantha E. M. Munroe, Francesca A. McInerney, Greg R. Guerin, Jake W. Andrae, Nina Welti, Stefan Caddy-Retalic, Rachel Atkins, Ben Sparrow

**Affiliations:** 1 School of Biological Sciences, The University of Adelaide, Adelaide, South Australia, Australia; 2 Terrestrial Ecosystem Research Network (TERN), University of Adelaide, Adelaide, South Australia, Australia; 3 School of Physical Sciences and the Sprigg Geobiology Centre, The University of Adelaide, Adelaide, South Australia, Australia; 4 CSIRO Agriculture and Food, Urrbrae, South Australia, Australia; 5 School of Life and Environmental Sciences, University of Sydney, New South Wales, Sydney, Australia; Chinese Academy of Forestry, CHINA

## Abstract

Numerous studies have analysed the relationship between C_4_ plant cover and climate. However, few have examined how different C_4_ taxa vary in their response to climate, or how environmental factors alter C_4_:C_3_ abundance. Here we investigate (a) how proportional C_4_ plant cover and richness (relative to C_3_) responds to changes in climate and local environmental factors, and (b) if this response is consistent among families. Proportional cover and richness of C_4_ species were determined at 541 one-hectare plots across Australia for 14 families. C_4_ cover and richness of the most common and abundant families were regressed against climate and local parameters. C_4_ richness and cover in the monocot families Poaceae and Cyperaceae increased with latitude and were strongly positively correlated with January temperatures, however C_4_ Cyperaceae occupied a more restricted temperature range. Seasonal rainfall, soil pH, soil texture, and tree cover modified proportional C_4_ cover in both families. Eudicot families displayed considerable variation in C_4_ distribution patterns. Proportional C_4_ Euphorbiaceae richness and cover were negatively correlated with increased moisture availability (i.e. high rainfall and low aridity), indicating they were more common in dry environments. Proportional C_4_ Chenopodiaceae richness and cover were weakly correlated with climate and local environmental factors, including soil texture. However, the explanatory power of C_4_ Chenopodiaceae models were poor, suggesting none of the factors considered in this study strongly influenced Chenopodiaceae distribution. Proportional C_4_ richness and cover in Aizoaceae, Amaranthaceae, and Portulacaceae increased with latitude, suggesting C_4_ cover and richness in these families increased with temperature and summer rainfall, but sample size was insufficient for regression analysis. Results demonstrate the unique relationships between different C_4_ taxa and climate, and the significant modifying effects of environmental factors on C_4_ distribution. Our work also revealed C_4_ families will not exhibit similar responses to local perturbations or climate.

## Introduction

C_3_ and C_4_ plants have distinct geographical distributions in large part due to differences in anatomy, physiology, and biochemistry [1]. Under conditions such as high heat or hypersalinity, C_3_ plants experience high levels of photorespiration [oxygen fixation by rubisco; 2–4]. Consequently, C_3_ plants may not thrive in hot or dry environments. C_4_ photosynthesis is distinguished by a series of biochemical and anatomical adaptations that concentrate and isolate CO_2_ with rubisco, helping to eliminate photorespiration and increase nutrient and water-use efficiency [5,6]. As a result, C_4_ plants can dominate warm environments and are essential to the development of tropical and subtropical savannahs, grasslands, and shrublands [5,7].

Broad patterns in C_3_ and C_4_ cover are predominantly estimated using temperature and precipitation models [8,9]. However, the relative importance of different environmental factors is still debated. As a result, numerous approaches have been developed to estimate C_4_ distribution. The leading method used to predict C_3_:C_4_ grass cover is the physiological temperature crossover model [10,11]. This model incorporates the antagonistic effects of increasing temperature and rising CO_2_ levels on C_3_ and C_4_ plants. Increases in CO_2_ levels are expected to favour C_3_ species, potentially leading to increased C_3_ cover [12–14], while rising temperatures should favour C_4_ species [15]. The crossover approach predicts that at modern CO_2_ levels_,_ the photosynthetic yields (i.e. light use efficiencies) of C_4_ grasses surpasses those of C_3_ grasses at a crossover temperature of approximately of 22°C [10,11,16]. However, more recent research has challenged the underlying physiological assumptions of this approach, arguing that CO_2_ levels have a reduced influence relative to other environmental factors [15,17]. Alternative models to calculate C_4_ cover include summer maximum temperatures [18] and seasonal rainfall patterns [19–21]. The method deemed to be the most accurate often differs between regions and the types of data used to develop the model. Therefore, there remains uncertainty on how to best predict C_4_ abundance, and how C_4_ and C_3_ species respond to environmental change.

Most C_4_ cover~climate models are designed to estimate C_4_ grass distribution. Few investigations have considered how additional C_4_ taxa vary in their particular relationship to climate, a critical feature in anticipating responses to environmental change. Although most C_4_ plants are indeed grasses (4500 species), C_4_ lineages are also found among sedges (1500 species) and eudicots [1200 species; 5]. Work that is available has revealed distinct C_4_ taxa exhibit disparate geographical trends [22,23]. For example, Wang and Ma [24] used flora survey data to examine C_4_ distribution in China and found that while C_4_ grass occurrence was primarily associated with changes in precipitation, the distributions of C_4_ chenopods and sedges were associated with changes in aridity and temperature, respectively. These studies demonstrate different models and approaches should be developed and applied across distinct lineages to provide the most accurate estimates of vegetation abundance.

Methods to predict C_4_ species distribution assume that species occurrence and cover are shaped by their physiological responses to climate. However, local environmental factors can significantly modify C_4_ distribution, influence local competitive dynamics, and inhibit or maintain C_3_ and C_4_ coexistence [25–27]. For example, Griffith, Anderson [28] determined the proportional cover of C_4_ grass in over 40,000 vegetation plots in North America. The best models to describe C_3_:C_4_ grass distribution included temperature, and precipitation, but also local environmental factors such as tree cover and fire. Local factors, such as soil nitrogen supply, have also been shown to reverse the responses of C_3_ and C_4_ plants to atmospheric CO_2_ concentrations [29]. Investigating the combined effects of climate and local factors on C_4_ distribution at large-scales is essential to accurately predicting C_4_ abundance now and into the future.

Large-scale studies of C_4_ occurrence and cover that consider both climate and other environmental factors have been limited due to a lack of data. Since 2009, the Terrestrial Ecosystem Research Network (TERN) has collected plot-based environmental data across all major biomes and dryland habitats in Australia. Using a consistent point-intercept approach, TERN systematically surveys one-hectare plots to determine the relative cover of plant taxa. In addition, the TERN plant sample library can be used to identify the photosynthetic pathway of unassigned species via stable carbon isotope analysis [30,31]. By combining these two novel resources, TERN’s monitoring program provides a rare opportunity to investigate the proportional cover and richness of different C_4_ lineages at a large scale. This work is particularly valuable for Australia, where only a few studies have examined the distribution of C_4_ grasses and sedges [32–35], and no studies have surveyed the distribution of C_4_ plants in other taxa.

In this study, we used TERN vegetation surveys to compare proportional vegetation cover and richness of C_4_ species in 14 plant families in Australia. We statistically assessed correlations between proportional C_4_ richness and cover with climate and edaphic variables in the four most common and abundant families: Poaceae, Cyperaceae, Chenopodiaceae and Euphorbiaceae. Specifically, we asked how proportional C_4_ plant richness and cover (relative to C_3_) responds to changes in climate and local environmental factors, and if this response was consistent among families. The analysis presented here sheds new light on how C_4_ occurrence and cover responds to particular climate variables, how local environmental factors modify C_4_ abundance, and whether responses are consistent among broad taxonomic groups. This work will inform improved strategies to estimate C_4_ abundance, and increase understanding of the impacts local conditions have on different C_4_ taxa.

## Materials and methods

### Data collection

Proportional C_4_ vegetation richness and cover (relative to combined C_3_ and C_4_ cover and richness) for each family were determined at 541 one-hectare plots systemically surveyed by TERN between 2011 and 2017. TERN plots are not surveyed on an annual basis. Instead, plots have typically been surveyed once, with an intention to revisit at least once per decade. Surveys occur between February to November. The season of survey is chosen to be the most appropriate for a given system to capture species diversity and provide the best chance of accurate species identification. Most plots are located within the Australian rangelands, which are characterised by weathered features, old and typically infertile soils, highly variable rainfall, and variable plant communities. Plot locations are selected to avoid major anthropogenic influences (such as roads, cattle yards, fences, bores, etc.) or sources of anthropogenic disturbance (e.g. livestock grazing). Surveyed vegetation types include woodlands and savannahs, tussock and hummock grasslands, and shrublands.

Plots (1 ha, 100 x 100 m) are permanently established sites located in a homogenous area of terrestrial vegetation. Transects (10 x 100 m long) were laid out within each plot in a 5 x 5 grid pattern. Parallel transects were spaced 20 meters apart. Species were recorded at each point (1 m) within the transect, resulting in 1010 survey points per plot. Point-intercept data were used to calculate proportional C_4_ vegetation cover and presence/absence data were used to calculate proportional C_4_ species richness (see statistical analysis). Ground observers vouchered all species present within each plot and vouchers were identified at major herbaria across Australia. Full survey protocols are detailed in the TERN Ausplots Rangelands manual [36,37].

### Statistical analysis

Data were analysed in the R statistical environment [38]. Point-intercept data were imported using the ‘ausplotsR’ package [39,40]. Species cover (%) was calculated from point-intercepts at each TERN plot using the ‘species_table’ function. The ‘species_table’ function calculates species cover (%) as the total number of times a species was detected or ‘counted’ across all transects, divided by the total number of transect points in the plot (1010), and multiplied by 100. If more than one survey was available, we calculated the average species cover for each plot. Species were assigned a photosynthetic pathway using [41]. The cover values of species with the same pathway were summed to determine the total C_4_ and C_3_ plant cover in each family at each plot. Finally, proportional C_4_ cover was calculated at each plot for each family as a proportion of C_3_ and C_4_ species cover by:

$$Proportional\;C_{4}\;cover=C_{4}\;species\;cover/(C_{4}\;species\;cover+C_{3}\;species\;cover)$$ (1)

Plot C_4_ and C_3_ richness was calculated as the number of unique C_4_ and C_3_ taxa at the species level that were detected across all transects in a plot. Proportional C_4_ richness for each family was calculated as a proportion of the combined species richness of C_3_ and C_4_ species by:

$$Proportional\;C_{4}\;species\;richness=C_{4}\;richness/(C_{4}\;richness+C_{3}\;richness)$$ (2)

We decided to examine C_4_ richness and cover as a proportion of total C_3_ and C_4_ species richness and cover to prevent our analysis from being influenced by factors that affect plant abundance. Much of Australia has a highly arid climate with limited plant cover, and these habitats can’t be easily directly compared to temperate or wet-tropical regions.

To investigate the unique cover patterns of C_4_ species in different plant families, proportional C_4_ richness and cover were regressed against climatic and local environmental parameters that are considered potential drivers of C_4_ plant distribution [5,28,42]. Climate variables included mean annual temperature (MAT), mean annual precipitation (MAP), mean January minimum and maximum temperature [18,33], January precipitation, mean annual aridity index (precipitation/potential evaporation), C_4_ growing season water availability [see desription below; 19,32], and a variable generated to represent the Collatz, Berry [11] crossover temperature model (see below). Local edaphic variables included soil sand and clay content (%), pH, and available water capacity (the amount of water soil can store that is available to plants, AWC). Edaphic values were averaged over a soil depth of 0 to 15 cm. We also included tree cover (%), which was calculated as the total species cover (%) of trees equal to or taller than 5 m at each plot. Climate data were based on 1970–2018 records and were sourced from the Australian Gridded Climate Data set (Bureau of Meteorology, accessed through the TERN Data Discovery Portal). The aridity index used data spanning 1976–2005 and was sourced from Harwood, Donohue [43]. Soil variables were accessed from the Soil and Landscape Grid of Australia [44].

C_4_ growing season water availability (hereafter, seasonal water availability or SWA) was calculated according to Murphy and Bowman [32]. This approach determines the proportion of precipitation that occurs during C_3_ and C_4_ growing seasons as defined by temperature and was calculated by:

$$SWA=\frac{Precipitation\;in\;C_{4}\;growing\;season}{\left(Precipitation\;in\;C_{4}\;growing\;season+precipitation\;in\;C_{3}\;growing\;season\right)}$$ (3)

To apply the Collatz, Berry [11] crossover temperature approach as consistently as possible, we regressed the mean annual proportion of C_4_ favoured months rather than the mean absolute number of C_4_ favoured months. A given month was determined to favour C_3_ growth when the mean daytime temperature was ≤ 22°C and precipitation was ≥ 25 mm. A given month was determined to favour C_4_ growth when the mean daytime temperature was > 22°C and precipitation was ≥ 25 mm. As previously mentioned, most of Australia has a highly arid climate and large areas of the country receive < 25 mm of precipitation per month. Therefore, comparing the absolute number of C_4_ favoured months in each plot would have confounded comparisons between dry and wet habitats.

To investigate the partial effects of different climate and local environmental variables, we considered several possible parametric and non-parametric approaches. Because our data are proportional (i.e. range from 0 to 1 and included true values of 0 and 1) and were derived from discrete counts (i.e. the number of C_4_ plants out of the total number of plants in the transect), a logistic regression with a weighted response variable was determined an appropriate method to explore the relationship between proportional C_4_ plot values and climate and local variables [45]. Models were constructed using the *glm* function. Strong covariance among variables or the inclusion of too many variables can lead to overfitting [46]. Therefore, models were limited to variables that had Pearson pairwise correlations < 0.7 and a maximum of five predictors. Although we acknowledge interactions are probable in these complex systems, preliminary work showed that our sample size was not large enough to support interactions. Models were compared using a backwards, step-wise comparison process between each model using quasi-Akaike information criterion (QAIC) to account for over-dispersion. Spatial autocorrelation was tested using Moran’s *I* [47]. In cases where we identified significant (p<0.05) spatial autocorrelation, a spatial autocovariate term was included as a fixed covariate in each model. Autocorrelation was detected in Poaceae and Chenopodiaceae regressions, therefore spatial autocovariate terms were included as fixed covariates in Poaceae and Chenopodiaceae models. The spatial autocovariate terms were calculated as the distance-weighted mean of neighbouring proportional C_4_ values. The terms were calculated using the R package “spdep” [48]. A pseudo- R^2^ (McFadden’s R^2^) was used as a measure of explained variation [49]. To determine the relative importance of each predictor in the best fit models, each predictor was removed from the best fit model (i.e. a leave-one-out approach) to compare the change in QAIC to the full model. Models were visualised using the “visreg” package in R [50].

## Results

### Proportional C_4_ cover and richness

Using Munroe, McInerney [41], we determined the photosynthetic pathway of 2484 of the 2605 species identified within TERN plots. Most unassigned species were rarely encountered (i.e. only recorded at one point in a single plot) and thus had a limited effect on analysis. Of the 2484 assigned species, 347 (13.9%) were C_4_ species and 2101 were C_3_ (84.5%). The remaining species were C_3_-CAM (18), CAM (7), C_3_-C_4_ (7), and C_4_-CAM (4). C_4_ species were distributed amongst 14 families and 85 genera (S1 Table). To enable consistent comparisons with previous work, we evaluated Chenopodiaceae independent of Amaranthaceae [e.g. 24,51]. C_3_-CAM, CAM, C_3_-C_4_, and C_4_-CAM species were excluded from statistical analysis.

**Fig 1:**
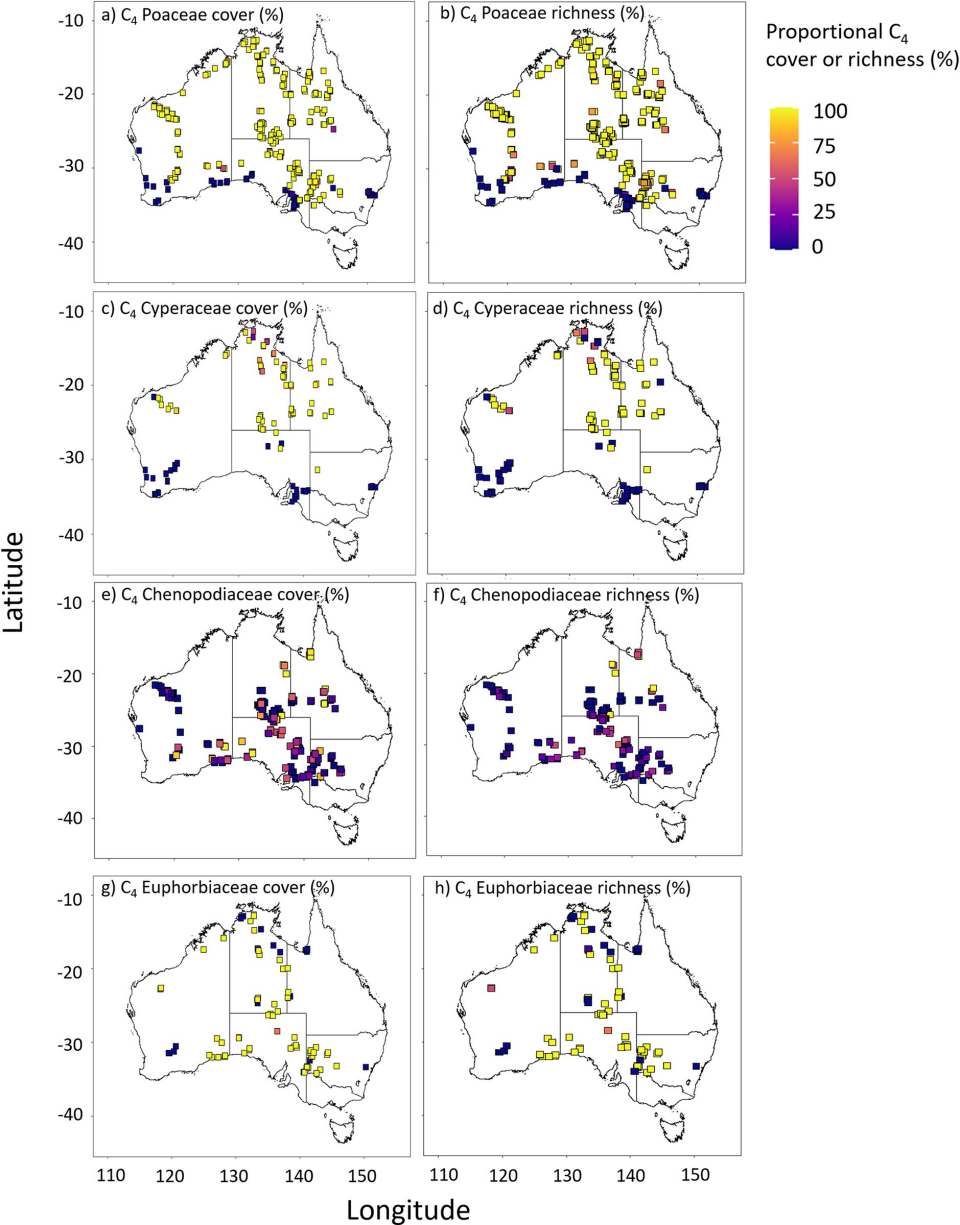
Proportional C_4_ cover and richness at TERN monitoring plots. (a, b) Poaceae, (c, d) Cyperaceae, (e, f) Chenopodiaceae, and (g, h) Euphorbiaceae.

C_4_ plants were detected in 451 plots (85.7%). Proportional C_4_ richness ranged from 0 to 88% (24.8% ±19.1), where 81% of plots had ≥ 5% C_4_ richness, and 16% of plots had ≥ 50% C_4_ richness. Proportional C_4_ cover ranged from 0 to 98% (36.5% ± 30.7%, mean ± standard deviation). 72% of TERN plots had ≥ 5% proportional C_4_ cover and 35% of plots had ≥ 50% proportional C_4_ cover. We calculated proportional C_4_ richness and cover at TERN plots for all 14 families in which C_4_ species were identified (S1 Data). Proportional C_4_ richness and cover in Aizoaceae, Amaranthaceae, Cyperaceae, Poaceae, and Portulacaceae increased along a south to north trajectory (Figs 1 and S1 File). Although not tested for directly, these trends indicate C_4_ cover in these families increased with increases in temperature, C_4_ growing season, and summer rainfall. Proportional C_4_ cover and richness in Boraginaceae and Zygophyllaceae was also typically higher in northern Australia. However, C_3_ species in these families were also common in some northern plots. The remaining eudicot families exhibited no obvious geographical or climate-based trends in proportional C_4_ distribution. C_3_ Asteraceae species were found distributed across TERN plots, with only one C_4_ species being detected in western Australia in two plots. Both C_3_ and C_4_ Cleomaceae were found in northern subtropical plots. C_4_ Euphorbiaceae and Chenopodiaceae were found in a wide range of climates, including the relatively cool, temperate areas of southern Australia. There were sufficient data for logistic regression analysis of Poaceae, Cyperaceae, Chenopodiaceae, and Euphorbiaceae. For all other families, species were detected in too few plots (< 50) or had too little cover (< 1%) to conduct effective quantitative analysis.

### Poaceae

**Fig 2:**
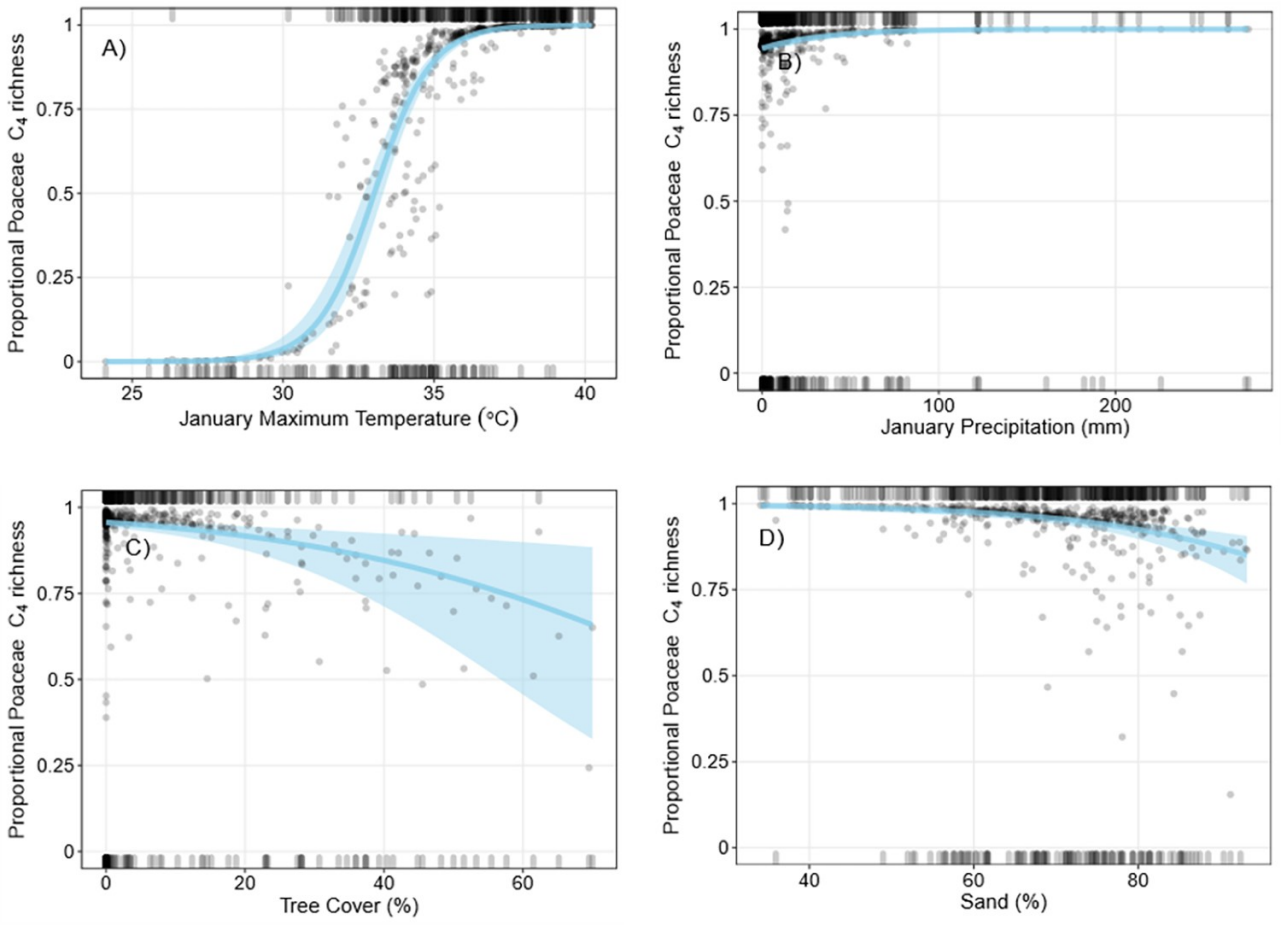
Binomial logistic regressions of proportional C_4_ Poaceae richness. Grey points are partial residuals, blue lines and shaded bands are predicted outcomes of the regression and 95% confidence intervals respectively, and rugs were drawn to indicate observations with positive residuals (top of the plot) or negative residuals (bottom of the plot). For each plot (A-D), independent variables not depicted on the x-axis are held constant at their median value.

**Fig 3:**
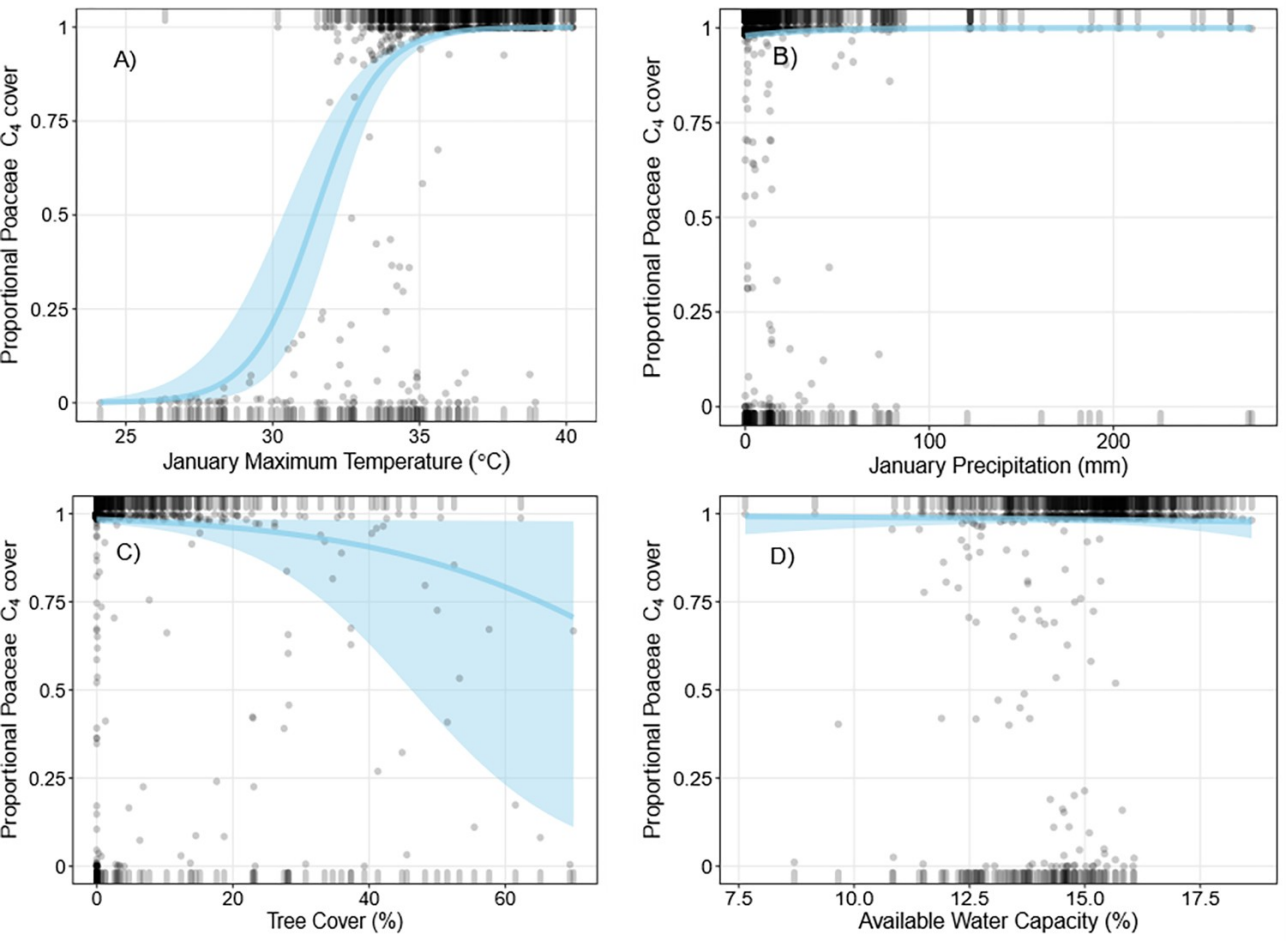
Binomial logistic regressions of proportional C_4_ Poaceae cover. Grey points are partial residuals, blue lines and shaded bands are predicted outcomes of the regression and 95% confidence intervals respectively, and rugs were drawn to indicate observations with positive residuals (top of the plot) or negative residuals (bottom of the plot). For each plot (A-D), independent variables not depicted on the x-axis are held constant at their median value.

Mean proportional C_4_ Poaceae richness was 82.8% (± 34.0) and mean proportional C_4_ Poaceae cover was 83.9% (± 34.9%). Most plots containing Poaceae were characterised by either 0 or 100% proportional C_4_ Poaceae richness and cover (Figs 2 and 3, SI Appendix 3). Mean January maximum temperature, January precipitation, tree cover, and sand (%) were the best predictors of proportional C_4_ Poaceae richness (Table 1). Proportional C_4_ Poaceae richness increased with January maximum temperature (Fig 2). Higher January precipitation and low tree cover were correlated with comparatively small increases in proportional C_4_ Poaceae richness. Sand (%) also had minor modifying effect on proportional C_4_ Poaceae richness, where increased sand (%) were associated with decreased proportional richness. QAIC comparisons using our leave-one-out approach indicated January maximum temperature was the most important predictor of C_4_ grass richness trends, while tree cover was of least importance in the model (S2 Table).

**Table 1:** Binomial logistic regression model selection results of proportional C_4_ richness for Poaceae, Cyperaceae, Chenopodiaceae, and Euphorbiaceae.

Family	Model	QAIC	ΔQAIC	R^2^
Poaceae	Jan. Max + Jan. Precip + sand + Tree Cover	448.1	0.00	0.67
	Jan. Max + Jan. Precip + sand + Tree Cover+AWC	448.4	0.26	0.67
	Jan. Max + Jan. Precip + sand + Tree Cover+pH	450.0	1.85	0.67
Cyperaceae	Collatz + pH + Tree Cover*	100.4	0.00	0.70
	Collatz + pH + Tree Cover + clay	101.1	0.71	0.70
	Collatz + pH + Tree Cover + sand	101.4	1.01	0.70
	Collatz + pH + Tree Cover + Jan. Precip	102.3	1.91	0.70
Chenopodiaceae	Jan. precip + sand*	484.5	0.00	0.09
	Jan. precip + sand + AWC	486.3	1.78	0.09
	Jan. precip + sand + MAT	486.3	1.81	0.09
	Jan. precip + sand + pH	486.5	1.95	0.09
	Jan. precip + sand + Jan. Min.	486.5	1.96	0.09
	Jan. precip + sand + SWA	486.5	1.97	0.09
	Jan. precip + sand + Jan.Max	486.5	1.98	0.09
	Jan. precip + sand + Tree Cover	486.5	1.98	0.09
	Jan. precip + sand + Collatz	486.5	2.00	0.09
Euphorbiaceae	AWC + MAP+ SWA	181.2	0.00	0.19
	AWC+MAP+Jan. Min.	119.3	0.00	0.18
	AWC + MAP+MAT	120.2	0.93	0.18
	AWC + MAP+Collatz	120.4	1.13	0.17
	AWC + MAP+Jan. Min.+pH	120.5	1.27	0.19
	AWC + MAP+pH	120.6	1.39	0.16
	AWC + MAP+MAT+pH	120.9	1.61	0.19
	AWC + MAP+Jan. Max. +SWA	121.0	1.76	0.19
	AWC + MAP+clay+Jan. Min. +pH	121.1	1.80	0.19
	AWC + MAP+clay+SWA	121.2	1.95	0.19
	AWC + MAP	121.2	1.96	0.15
	AWC + MAP+sand+SWA	121.3	1.99	0.19

Models were ranked using QAIC values, ΔQAIC is the difference between the model’s QAIC and the lowest QAIC of the candidate set. Only models with a ΔQAIC <2 are shown. The best fit models are marked by an asterisk (*). R^2^ is the McFadden’s R^2^ value of each model. Spatial autocovariate terms were included as fixed covariates in the Poaceae and Chenopodiaceae models. Predictor variables are mean annual temperature (MAT), mean annual precipitation (MAP), mean January minimum temperature (Jan. Min), mean January maximum temperature (Jan. Min), January precipitation (Jan. Precip), mean annual aridity index (Aridity), season water availability (SWA), mean annual proportion of C_4_ favoured months (Collatz) soil sand and clay content (%, sand, clay), pH, and available water capacity (AWC).

Mean January maximum temperature, January precipitation, AWC, and tree cover were the best predictors of proportional C_4_ Poaceae cover (Tables 2 and S2). January maximum temperature was positively correlated with increases in proportional C_4_ Poaceae cover (Fig 3). Low tree cover, increased January precipitation, and lower AWC was correlated with small increases in proportional C_4_ Poaceae cover. However, only January maximum temperature, January precipitation, and tree cover were consistently included in models with a QAIC<2; the removal of AWC had little effect on the overall R^2^ and QAIC values (S2 Table). QAIC comparisons using our leave-one-out approach also indicated January maximum temperature was the most important predictor of C_4_ grass cover trends (S2 Table). Both the proportional C_4_ grass cover and richness models predicted indicated that below 30°C January maximum temperature, C_3_ grasses dominated survey plots (>80% C_3_), while at plots with January maximum temperatures above 33°C, C_4_ grasses dominated (>80% C_4_). Temperatures between 30°C and 33°C supported mixed C_4_/C_3_ grass plots. There was a significant positive correlation between proportional C_4_ Poaceae richness and cover (Spearman’s rank correlation, ρ = 0.81, P<0.05, S2 File). Visual inspection of partial residuals indicated we achieved a better model fit for proportional C_4_ Poaceae richness than cover.

**Table 2:** Logistic regression model results of proportional C_4_ cover for Poaceae, Cyperaceae, Chenopodiaceae, and Euphorbiaceae.

Family	Model	QAIC	ΔQAIC	R^2^
Poaceae	AWC + Jan. Max + Jan. Precip + Tree cover*	483.8	0.0	0.68
	Jan. Max + Jan. Precip + Tree cover	484.7	0.9	0.68
	AWC+ Jan. Max + Jan. Precip + Tree cover+sand	484.9	1.1	0.68
	AWC+Jan. Max + Jan. Precip + Tree cover+MAP	485.1	1.3	0.68
	AWC+Jan. Max + Jan. Precip + Tree cover + pH	485.2	1.3	0.68
	AWC+MAT+ Jan. Max + Jan. Precip + Tree cover	485.6	1.7	0.68
	AWC+Aridity + Jan. Max + Jan. Precip + Tree cover	485.7	1.8	0.68
	AWC+clay+ Jan. Max + Jan. Precip + Tree cover	485.7	1.9	0.68
Cyperaceae	Jan. Min. + pH + Tree Cover+ Jan. Precip*	210.4	0.00	0.85
	Jan. Min. + pH + Tree Cover+ Jan. Precip+sand	211.9	1.5	0.85
Chenopodiaceae	Jan. Max + pH + sand + Tree cover*	322.0	0.00	0.15
	pH + sand + Tree cover	322.6	0.58	0.14
Euphorbiaceae	AWC + Aridity + Jan. Max + sand+ SWA	181.2	0.00	0.55

Models were ranked using QAIC values, ΔQAIC is the difference between the model’s QAIC and the lowest QAIC of the candidate set. Only models with a ΔQAIC <2 are shown. The best fit models are marked by an asterisk (*). R^2^ is the McFadden’s R^2^ value of each model. Spatial autocovariate terms were included as fixed covariates in the Poaceae and Chenopodiaceae models. Predictor variables are mean annual temperature (MAT), mean annual precipitation (MAP), mean January minimum temperature (Jan. Min), mean January maximum temperature (Jan. Min), January precipitation (Jan. Precip), mean annual aridity index (Aridity), season water availability (SWA), mean annual proportion of C_4_ favoured months (Collatz) soil sand and clay content (%, sand, clay), pH, and available water capacity (AWC).

### Cyperaceae

**Fig 4:**
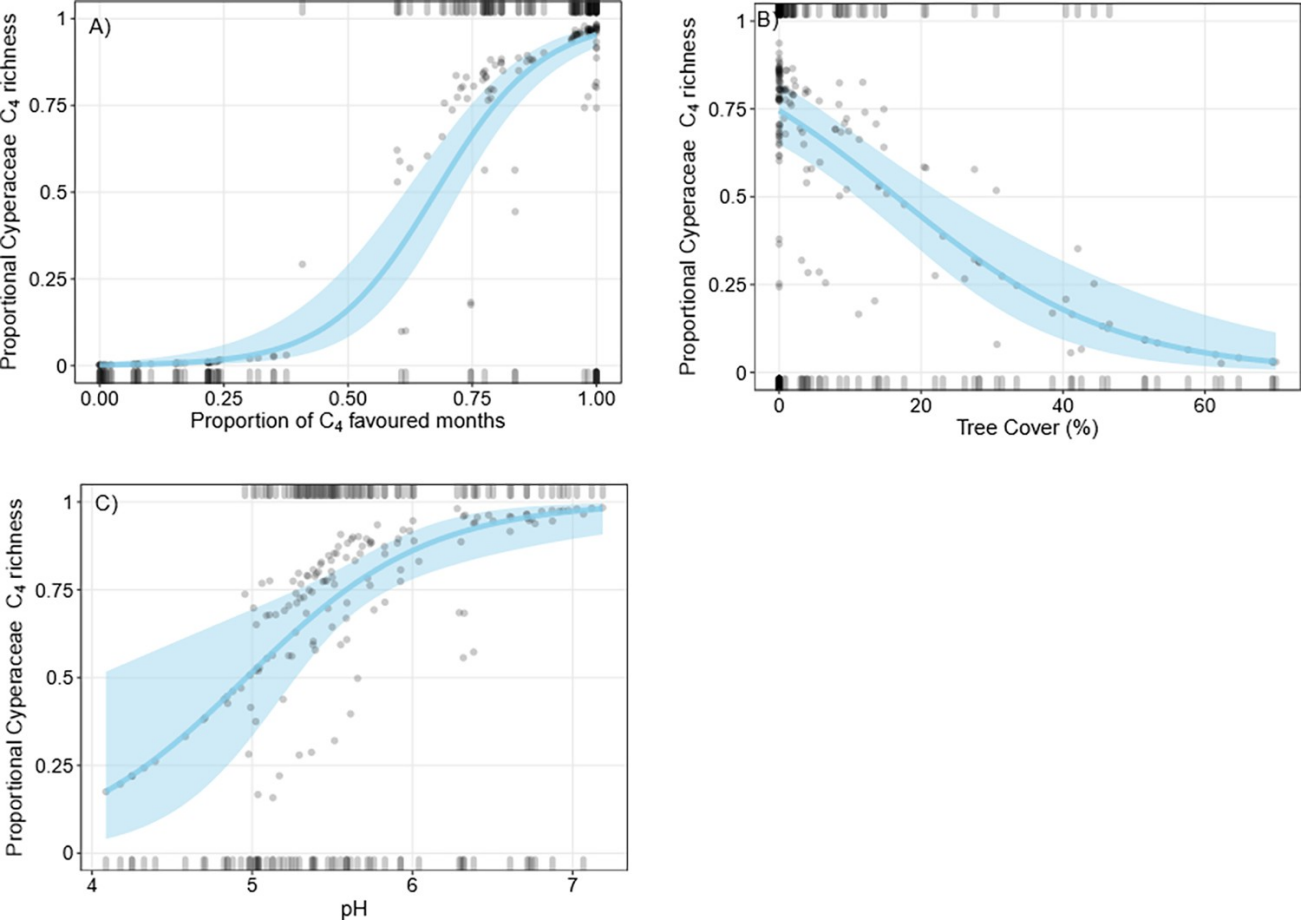
Binomial logistic regressions of proportional C_4_ Cyperaceae richness. Grey points are partial residuals, blue lines and shaded bands are predicted outcomes of the regression and 95% confidence intervals respectively, and rugs were drawn to indicate observations with positive residuals (top of the plot) or negative residuals (bottom of the plot). For each plot (A-C), independent variables not depicted on the x-axis were held constant at their median value.

**Fig 5:**
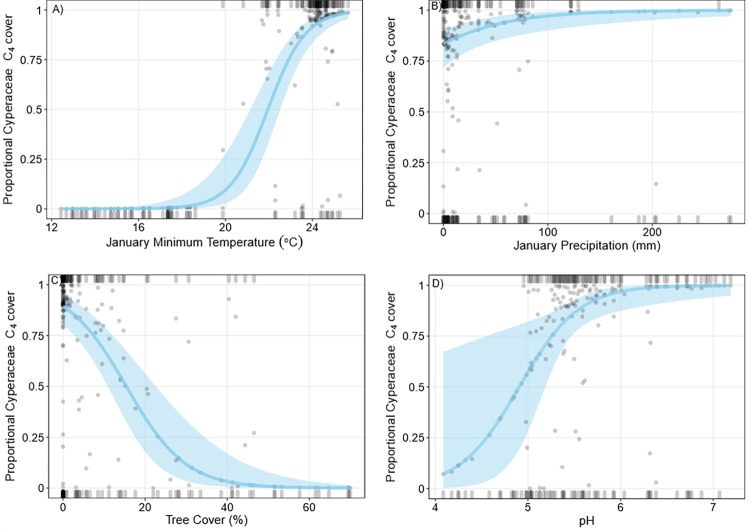
Binomial logistic regressions of proportional C_4_ Cyperaceae cover. Grey points are partial residuals, blue lines and shaded bands are predicted outcomes of the regression and 95% confidence intervals respectively, and rugs were drawn to indicate observations with positive residuals (top of the plot) or negative residuals (bottom of the plot). For each plot (A-D), independent variables not depicted on the x-axis were held constant at their median value.

Mean proportional C_4_ Cyperaceae richness was 52.8% (± 48.6) and mean proportional C_4_ Cyperaceae cover was 57.6% (± 48.2%). Most plots containing Cyperaceae were defined by bimodal values of 0 or 100% proportional C_4_ Cyperaceae cover and richness (Figs 4 and 5). The mean annual proportion of C_4_ favoured months [11], tree cover, and pH were included in the best fit model to predict predictors of proportional C_4_ Cyperaceae richness. Proportional C_4_ Cyperaceae richness was positively correlated with an increased proportion of C_4_ favoured months. Higher C_4_ Cyperaceae proportional richness was positively correlated with low tree cover and higher pH values (less acidic; Fig 4). QAIC comparisons using a leave-one-out approach indicated the proportion of C_4_ favoured months was the primary predictor of C_4_ Cyperaceae richness trends (S2 Table).

Mean January minimum temperature, January precipitation, tree cover and pH were included in the best fit model to predict proportional C_4_ Cyperaceae cover. Proportional C_4_ Cyperaceae cover was positively correlated with January minimum temperature and January precipitation (Fig 5). Low tree cover and higher pH were also positively correlated with increases in proportional C_4_ Cyperaceae cover. QAIC comparisons using a leave-one-out approach indicated January minimum temperature was the primary predictor of C_4_ Cyperaceae cover trends, followed by % tree cover (S2 Table). The proportional C_4_ Cyperaceae cover model predicted that below 20°C January minimum temperature, C_3_ Cyperaceae dominated survey plots (>80% cover), while at plots with January minimum temperatures above 23°C, C_4_ Cyperaceae dominated (>80% cover). Temperatures between 20°C and 23°C supported mixed C_4_/C_3_ Cyperaceae plots. There was a positive correlation between proportional C_4_ Cyperaceae richness and cover (Spearman’s rank correlation, ρ = 0.99, P<0.05; S2 File).

### Chenopodiaceae

**Fig 6:**
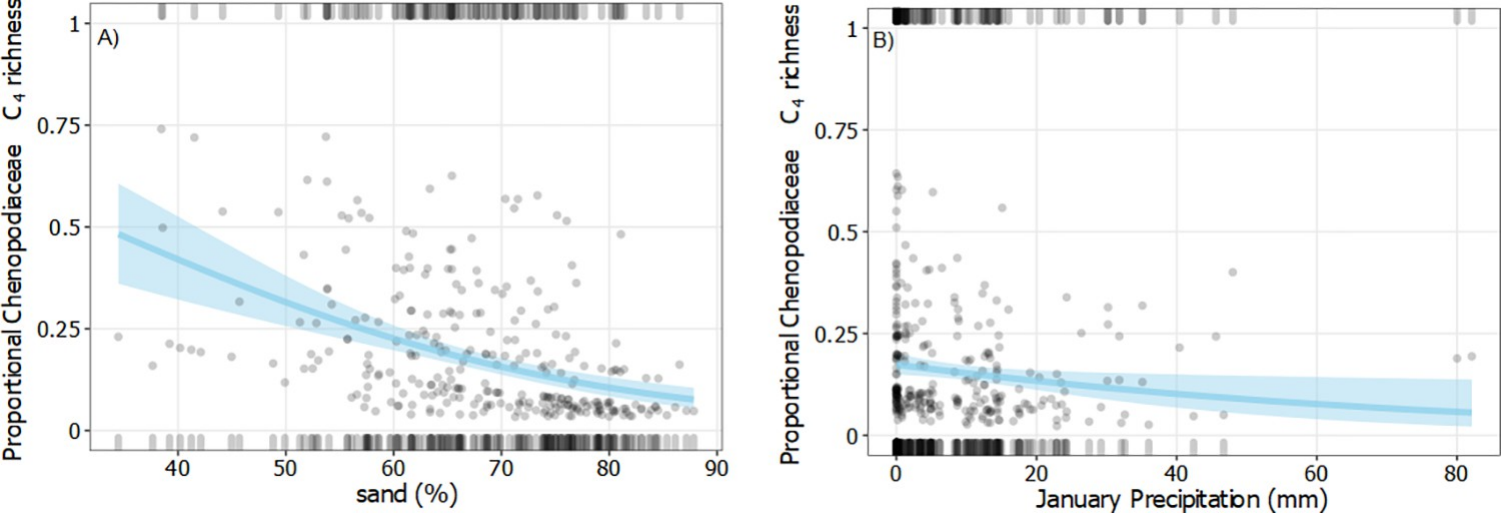
Binomial logistic regressions of proportional C_4_ Chenopodiaceae richness. Grey points are partial residuals, blue lines and shaded bands are predicted outcomes of the regression and 95% confidence intervals respectively, and rugs were drawn to indicate observations with positive residuals (top of the plot) or negative residuals (bottom of the plot). For each plot, independent variables not depicted on the x-axis were held constant at their median value.

**Fig 7:**
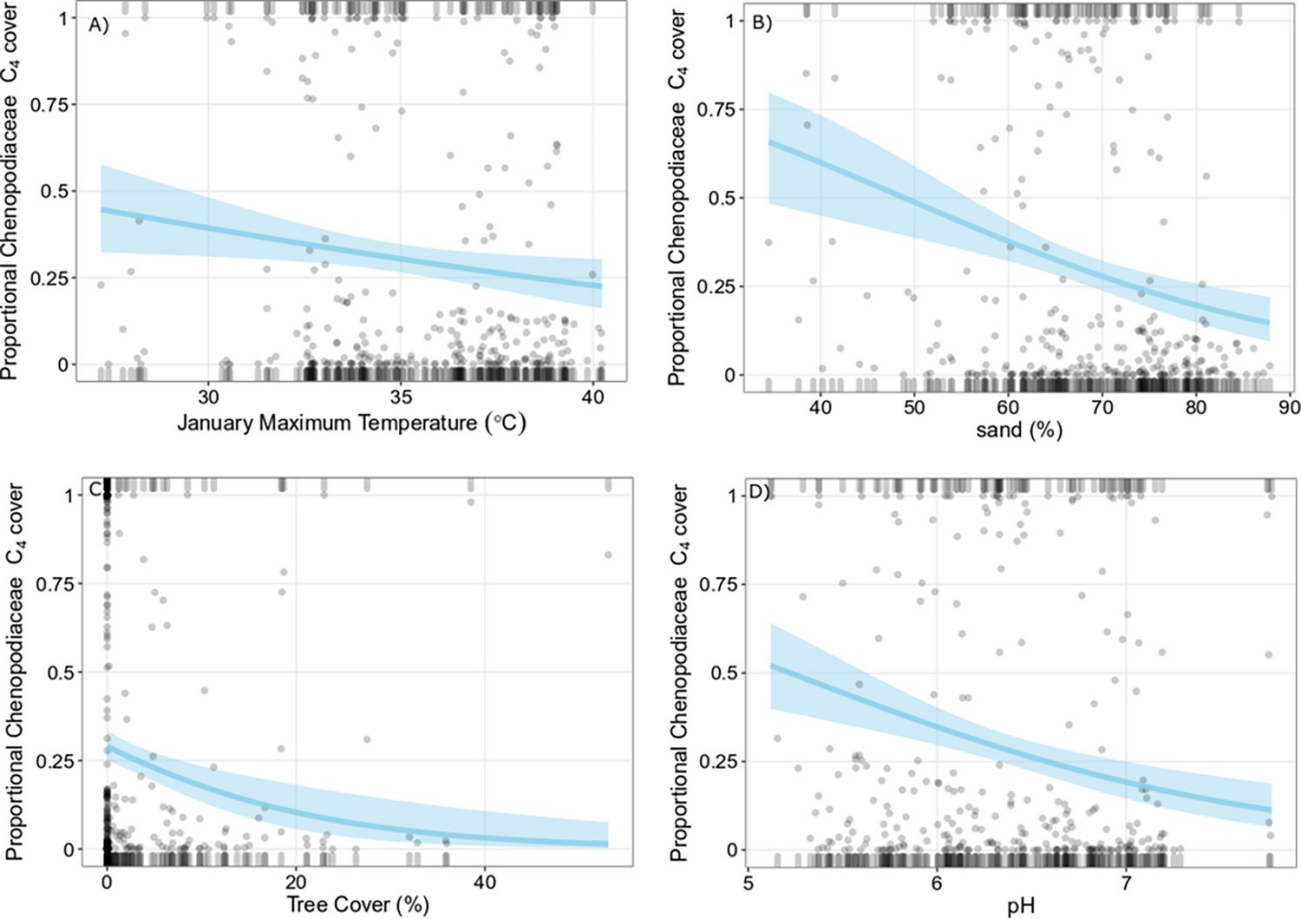
Binomial logistic regressions of proportional C_4_ Chenopodiaceae cover. Grey points are partial residuals, blue lines and shaded bands are predicted outcomes of the regression and 95% confidence intervals respectively, and rugs were drawn to indicate observations with positive residuals (top of the plot) or negative residuals (bottom of the plot). For each plot (A-D), independent variables not depicted on the x-axis were held constant at their median value.

Mean proportional C_4_ Chenopodiaceae richness was 15.8% (± 22.1) and mean proportional C_4_ Chenopodiaceae richness was cover was 19.0% (± 29.5). Proportional C_4_ Chenopodiaceae richness and cover was generally low (Figs 6 and 7). Plots that included Chenopodiaceae commonly contained a mix of C_3_ and C_4_ species. January precipitation and sand (%) were included in the best fit model to predict proportional C_4_ Chenopodiaceae richness (Table 1; Fig 6) but the model had low explanatory power and poor model fit. Lower sand content and January precipitation were weakly correlated with higher proportional C_4_ Chenopodiaceae richness (S2 Table). Mean January maximum temperature, tree cover, pH, and sand (%) were included in the best fit model to predict proportional C_4_ Chenopodiaceae cover (Table 2; Fig 7). However, examination of partial model residuals and R^2^ values indicates that even the best fit model performed poorly and had little explanatory power. Increased proportional C_4_ Chenopodiaceae cover was only weakly associated with decreasing tree cover, lower soil pH and sand content (S2 Table). There was a positive correlation between proportional C_4_ Chenopodiaceae richness and cover (Spearman’s rank correlation, ρ = 0.92, P<0.05; S2 File).

### Euphorbiaceae

**Fig 8:**
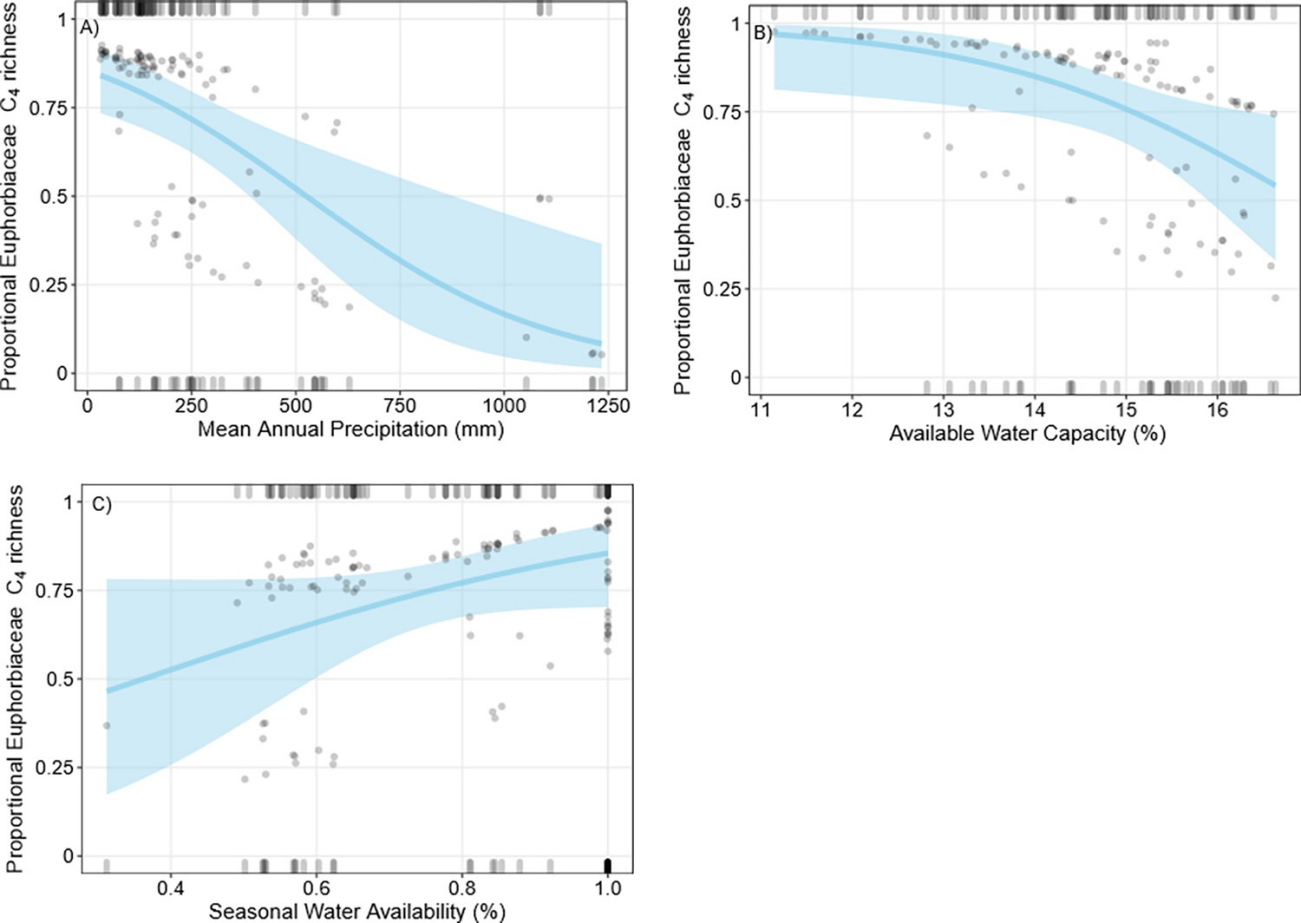
Binomial logistic regressions of proportional C_4_ Euphorbiaceae richness. Grey points are partial residuals, blue lines and shaded bands are predicted outcomes of the regression and 95% confidence intervals respectively, and rugs were drawn to indicate observations with positive residuals (top of the plot) or negative residuals (bottom of the plot). For each plot, independent variables not depicted on the x-axis were held constant at their median value.

**Fig 9:**
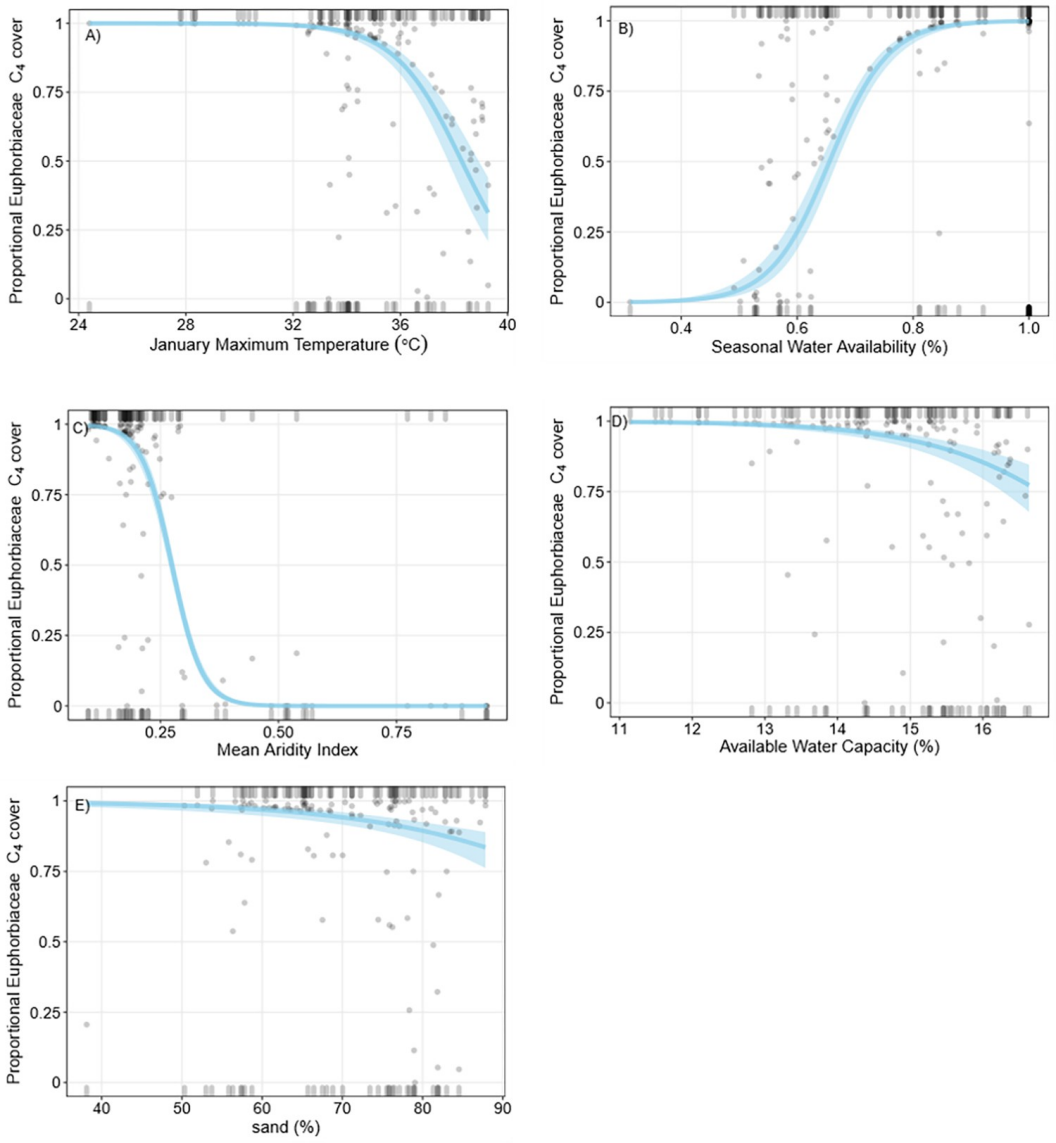
Binomial logistic regressions of proportional C_4_ Euphorbiaceae cover. Grey points are partial residuals, blue lines and shaded bands are predicted outcomes of the regression and 95% confidence intervals respectively, and rugs were drawn to indicate observations with positive residuals (top of the plot) or negative residuals (bottom of the plot). For each plot, independent variables not depicted on the x-axis were held constant at their median value.

Mean proportional C_4_ Euphorbiaceae richness was 69.1% (± 44.8%) and mean proportional C_4_ Euphorbiaceae cover was 68.5% (± 45.3). The total number of Euphorbiaceae species per plot was low (≤ 2), and they accounted for a small amount of the total vegetation cover at each plot (<1–20%). MAP, SWA, and AWC were included in the best fit model to predict proportional C_4_ Euphorbiaceae richness (Table 1). Proportional C_4_ Euphorbiaceae richness was negatively correlated with MAP, SWA and AWC (Fig 8), however the model fit and explanatory power of the C_4_ Euphorbiaceae richness logistic regression was relatively poor. SWA, sand (%), aridity, mean January maximum temperature, and AWC were included in the best fit model to predict proportional C_4_ Euphorbiaceae cover. Increased January maximum temperature, higher aridity index values (i.e. wetter conditions), higher AWC, and high soil sand content (%) were negatively correlated with proportional C_4_ Euphorbiaceae cover (Fig 9), while increased SWA was positively correlated with proportional C_4_ Euphorbiaceae cover. Leave-one-out QAIC comparisons indicated aridity and SWA were the most important predictors of proportional C_4_ Euphorbiaceae cover (S2 Table). There was a positive correlation between proportional C_4_ Euphorbiaceae richness and cover (Spearman’s rank correlation, ρ = 0.99, P<0.05; S2 File).

## Discussion

Our analysis of proportional C_4_ richness and cover revealed different families exhibit divergent responses to both climate and local environmental conditions. Although temperature was a key driver of proportional C_4_ monocot distribution, local environmental factors also had significant modifying effects on C_4_ richness and cover. Eudicot families displayed unique and sometimes contrasting C_4_ distribution patterns. While proportional C_4_ cover and richness in families such as Aizoaceae and Portulacaceae increased with latitude, suggesting there was a strong relationship with C_4_ in these families and temperature, climate had a limited apparent influence on proportional C_4_ Chenopodiaceae distribution. Cumulatively, our work demonstrates different C_4_ lineages will display diverse responses to climate, as well as local environmental variation, and that climate patterns alone cannot explain trends in C_4_ distribution.

Proportional C_4_ Poaceae cover and richness increased with increases in January maximum temperatures and summer rainfall. Our results are congruent with other studies from Australia and around the world which have similarly concluded summer temperatures are strongly correlated with absolute and proportional C_4_ grass cover and richness [18,33,35,52–54]. Our results are also consistent with von Fischer, Tieszen [18], who also found summer rainfall had a significant relationship with North American C_4_ biomass. Angelo and Daehler [54] also found precipitation had a weak but significant relationship with C_4_ grass cover along tropical elevation gradients in Hawaii. Interestingly, the adaptation of the Collatz, Berry [11] crossover approach was not the best model to explain C_4_ distribution in Poaceae. More importantly, it was difficult to apply the crossover approach consistently across Australia. Dry regions of Australia often receive less than the 25 mm of precipitation per month needed to assign plots a C_3_ or C_4_-dominate status. As a result, for large areas of Australia, a traditional crossover approach was not feasible. This demonstrates the inherent limitations of the crossover method in Australia. For these reasons, we argue this metric should be avoided when predicting C_4_ grass distribution in Australia, and in other highly arid regions.

Regression analysis also indicated that proportional C_4_ grass richness and cover was influenced by local environmental factors. Increased tree cover had a negative effect on proportional C_4_ Poaceae cover and richness, which may reflect the increased difficulty for all C_4_ plants to grow under shade [28,42,55–57]. Although regression analysis also indicated AWC was an important predictor of proportional C_4_ Poaceae cover, the overall impact of AWC is questionable and should be interpreted with caution given the poor fit of the data and the fact that its removal from the best fit model had a limited impact on R^2^ or QAIC values. It is possible that AWC, soil texture (i.e. sand content), and tree cover are acting as useful indicators of local water availability (where increased tree cover is typically associated with greater moisture availability and rainfall). Local water availability can have a substantial effect on C_3_:C_4_ competitive dynamics. Increased moisture availability, particularly in warm mesic habitats, may mitigate the benefits of increased water-use efficiency in C_4_ species [5] and provide C_3_ grasses with a competitive advantage, ultimately supporting species co-existence [26]. However, capturing the nuanced influence of these local factors requires further study at a finer scale.

Like Poaceae, C_4_ Cyperaceae cover was correlated with increases in summer temperatures and C_4_ growing season length. However, C_4_ Cyperaceae had a more restricted temperature range than C_4_ Poaceae, indicating C_4_ Poaceae can occupy relatively cooler climates. These results are consistent with Stock, Chuba [58], who found the transitional temperatures of proportional C_4_ Poaceae and Cyperaceae richness in South Africa were 23°C and 34°C, respectively. Our models provided limited evidence of a strong relationship between proportional C_4_ Cyperaceae cover or richness and rainfall. Similar to Poaceae, proportional C_4_ Cyperaceae cover increase slightly as January precipitation increased, but the overall effect was minor, and precipitation had no apparent influence on proportional C_4_ Cyperaceae richness. Previous work examining Cyperaceae occurrence in other countries has also found temperature is the strongest driver of proportional C_4_ Cyperaceae richness, with moisture availability having little impact [22,24,58,59]. The absence of moisture-C_4_ Cyperaceae richness correlations has been attributed to the large number of Cyperaceae that prefer wet habitats [23,24]. Our work supports the hypothesis that precipitation has a more limited impact on the relative occurrence of C_4_ sedges as compared to temperature.

The significant influence of pH on proportional C_4_ Cyperaceae richness and cover could suggest soil biochemistry plays a role in C_3_:C_4_ sedge dynamics. Alkaline soils are less soluble than acidic soils, which can limit nutrient availability [60,61]. The relative impact of alkaline-stress on C_4_ and C_3_ plants has not been widely explored. However, C_4_ plants are considered more resistant to stress and thus may be more tolerant of alkaline soils [5,62,63]. pH is often correlated with other important conditions including salinity and soil fertility, thus this trend may reflect several interacting factors not explicitly considered here [29,63,64]. Although the causal relationship between these factors remains unclear, our results suggest that local factors modify C_4_ sedge abundance and richness and should be explored more deeply in future work.

Large-scale evaluations of C_4_ eudicot richness and cover are uncommon. Our results show several C_4_ eudicot lineages, chiefly Aizoaceae, Amaranthaceae, and Portulacaceae, follow expected geographical trends, where proportional C_4_ richness and cover increase from South to North, potentially in response to increasing temperatures and summer rainfall. However, other eudicot families, such as Asteraceae and Cleomaceae, did not display any clear latitudinal trends, suggesting C_4_ cover in these families is not driven by climate variables, but more likely by local environmental factors.

Despite reports of correlation with aridity in other regions [22,24,51,65], none of the climate or local environmental variables we considered were strongly correlated with proportional C_4_ Chenopodiaceae richness or cover. A potentially critical factor we were unable to consider due to lack of data was soil salinity. The evolution of C_4_ photosynthesis has long been considered a pre-adaption to arid and saline habitats [1,66,67], although additional work indicates adaptations to saline soils may have actually promoted C_4_ evolution in Chenopodiaceae [68]. As a result, salinity may be a key controlling factor of the distribution patterns of all C_4_ species in Australia [69]. Additional local factors such as soil nutrient content and salinity should be incorporated in future studies of C_4_ eudicot distribution where possible. Moreover, while plot location selection procedures were designed to reduce anthropogenic influences on native richness and cover estimates, the C_3_/C_4_ patterns reported here are not necessarily in equilibrium. Historical long-term grazing has led to substantial changes in species composition in some areas. For example, chenopods have been lost from large areas of Australia due to overgrazing [70]. Therefore, the environmental trends in C_3_/C_4_ richness and cover observed in this study, or lack thereof in the case of chenopods, may be a partial reflection of human influences on the landscape.

Increased rainfall and moisture availability were negatively correlated with proportional C_4_ Euphorbiaceae cover and richness, suggesting C_4_ Euphorbiaceae were more prevalent in dryer, although not necessarily hotter, conditions. This may explain why we did not detect a clear latitudinal trend in proportional C_4_ Euphorbiaceae cover and richness, as compared to monocot and some other eudicot taxa. Rainfall seasonality also appeared important, where plots with proportionally high C_4_ Euphorbiaceae cover and richness were only found in areas with greater than 50% SWA. Similar to our findings, Stowe and Teeri [65] found summer pan evaporation was most closely associated with North American C_4_ Euphorbiaceae distribution. In Egypt, C_4_ *Euphorbia* also occurred mainly in arid environments [71]. As ours is one of the few studies to examine C_4_ Euphorbiaceae dynamics at a large scale, these results are useful in understanding what factors control their distribution, not only in Australia, but globally. However, because Euphorbiaceae were less common in TERN plots compared to the other primary taxa investigated here, these results should be interpreted carefully and warrant further investigation.

Plots containing Poaceae and Cyperaceae exhibited a rapid transition from C_3_ to C_4_ dominated plots, with few plots containing a mix of both pathways. The bimodal distributions observed here may reflect the relatively rapid geographic transition in temperature and summer to winter dominated rainfall in Australia, leaving a limited habitat range where both species can survive. This finding has significant implications, since the geographic location of this narrow threshold may be highly sensitive to climate change [35]. Griffith, Anderson [28] also noted this bimodal trend in C_3_ and C_4_ grass distribution in North America, and suggested these extremes were maintained by local disturbance, mainly fire, which can support dominant relationships of one group over the other. Fire-prone environments may favour increased C_4_ grass cover in Australia [72,73] however, we were unable to test for this possibility in this study due to lack of data. Anecdotal observations by ground observers at some temperate TERN plots that experience regular burning have noted greater C_4_ cover compared to undisturbed areas, but a more precise fire history for these areas is needed to validate these observations.

The proportional cover and richness of C_4_ species are rarely simultaneously evaluated, although C_4_ grass presence and richness is commonly used to validate C_4_ cover at large scales [7,11,19,35]. Proportional C_4_ richness and cover were positively correlated in each family, suggesting richness can be used to broadly support cover estimates. However, the strength of these correlations was largely driven by the bimodal distribution in some families. There was still considerable variance between proportional C_4_ Poaceae and Chenopodiaceae richness and cover at mixed C_3_/C_4_ plots. Although our analysis was only designed to explore the influence of different factors, our work nonetheless indicated our ability to predict proportional C_4_ monocot richness is more accurate compared to proportional C_4_ monocot cover. These findings suggest the disproportionate relationship between C_4_ cover and richness is due to differences in the ability of species to occupy versus dominate an area. For example, given both proportional C_4_ Poaceae richness and cover were predominantly influenced by January maximum temperature, differences in model fit are likely determined by the modifying effects of water availability and other local environmental factors. Overall, our results demonstrate that C_4_ cover and richness provide unique assessments of species distribution and responses to the environment and thus should be measured together whenever possible.

This study is the first to compare the influence of both climate and local ecology on C_4_ grass, sedge, and eudicot cover and richness at a continental scale in Australia. Our results make clear that broadscale as well as localised environmental factors have divergent impacts on C_4_ taxa. While monocot lineages generally followed expected temperature-driven trends, there was considerable variability among eudicots families. Quantifying these differences is critical to predicting C_4_ cover under different environmental scenarios, or estimating plant resilience to small-scale perturbations. Future work can leverage the TERN datasets presented here to investigate more fine-scale taxonomic patterns, such as the influence of C_4_ subtype on species distribution and their relationship to climate [74,75]. Moreover, as TERN continues to expand its plot network and range of local environmental data, it may soon be possible to investigate the influence of fire salinity on C_4_ at both local and national scales.

## Supporting information

S1 Table Total number of species recorded in TERN survey plots in each photosynthetic pathway belonging to families with C4 species. (DOCX)

S2 Table Binomial logistic regression model results of proportional C4 cover and richness analysis, and results of leave-one-out comparisons of the best fit models to predict proportional C4 cover and richness. (DOCX)

S1 Data Proportional C4 richness and cover at each TERN plot for all 14 families in which C4 species were identified. (XLSX)

S1 File Maps of proportional C4 cover and richness at TERN Monitoring plots for additional 10 families in which C4 species were identified. (DOCX)

S2 File Proportional C4 richness versus cover. (DOCX)
