# Supplementary material for: Plant families exhibit unique geographic trends in C4 richness and cover in Australia
Source: PLoS One. 2022 Aug 22;17(8):e0271603. doi: 10.1371/journal.pone.0271603 (PMC9394836; doi:10.1371/journal.pone.0271603)
Supplement: S1 Table — (DOCX) [file pone.0271603.s001.docx]

Supporting Information 1

Plant families exhibit unique geographic trends in C_4_ richness and cover in Australia

Samantha E.M. Munroe*^1,2^, Francesca A. McInerney^3^, Greg R. Guerin^1,2^, Jake W. Andrae^3^, Nina Welti^4^, Stefan Caddy-Retalic^1,5^, Rachel Atkins^3^, & Ben Sparrow^1,2^

^1^ School of Biological Sciences, The University of Adelaide, Adelaide, South Australia 5005, Australia

^2^ Terrestrial Ecosystem Research Network (TERN), University of Adelaide, Adelaide, South Australia 5005, Australia

^3^ School of Physical Sciences and the Sprigg Geobiology Centre, The University of Adelaide, Adelaide, South Australia 5005, Australia

^4^ CSIRO Agriculture and Food, Urrbrae, South Australia 5064, Australia

^5^ School of Life and Environmental Sciences, University of Sydney, Sydney NSW 2006 Australia

*corresponding author: [samantha.munroe@adelaide.edu.au](mailto:samantha.munroe@adelaide.edu.au)

S1: Total number of species recorded in TERN survey plots in each photosynthetic pathway belonging to families with C_4_ species. “U” is the number of unassigned species, “% C_4_” is the proportion of species in each family with the C_4_ pathway, “nplot” is the number of plots where C_4_ species were detected/number plots where either C_3_ or C_4_ species were detected.

| **Family** | **C_3_** | **C_4_** | **CAM** | **C_3_-CAM** | **C_3_-C_4_** | **C_4_-CAM** | **U** | **% C_4_** | **nplot** |
| --- | --- | --- | --- | --- | --- | --- | --- | --- | --- |
| **Aizoaceae** | 6 | 3 | 1 | 5 | 0 | 0 | 0 | 20 | 16/54 |
| **Amaranthaceae** | 24 | 11 | 0 | 0 | 3 | 0 | 0 | 29 | 17/97 |
| **Asteraceae** | 177 | 1 | 1 | 0 | 0 | 0 | 7 | <1 | 2/232 |
| **Boraginaceae** | 15 | 10 | 0 | 0 | 0 | 0 | 2 | 37 | 13/66 |
| **Caryophyllaceae** | 9 | 1 | 0 | 0 | 2 | 0 | 0 | 8 | 1/3 |
| **Chenopodiaceae** | 106 | 25 | 0 | 0 | 0 | 1 | 0 | 19 | 156/318 |
| **Cleomaceae** | 2 | 1 | 0 | 0 | 0 | 0 | 0 | 33 | 1/23 |
| **Cyperaceae** | 61 | 38 | 0 | 0 | 0 | 0 | 0 | 38 | 94/167 |
| **Euphorbiaceae** | 12 | 13 | 0 | 1 | 0 | 0 | 1 | 48 | 77/117 |
| **Molluginaceae** | 0 | 1 | 0 | 0 | 0 | 0 | 0 | 100 | 1/1 |
| **Nyctaginaceae** | 0 | 7 | 0 | 0 | 0 | 0 | 0 | 100 | 41/41 |
| **Poaceae** | 90 | 229 | 0 | 0 | 0 | 0 | 1 | 71 | 427/502 |
| **Portulacaceae** | 1 | 4 | 0 | 8 | 1 | 3 | 0 | 23 | 8/25 |
| **Zygophyllaceae** | 16 | 3 | 0 | 0 | 0 | 0 | 0 | 15 | 14/72 |
