# Supplementary material for: Plant families exhibit unique geographic trends in C4 richness and cover in Australia
Source: PLoS One. 2022 Aug 22;17(8):e0271603. doi: 10.1371/journal.pone.0271603 (PMC9394836; doi:10.1371/journal.pone.0271603)
Supplement: S2 Table — (DOCX) [file pone.0271603.s002.docx]

Supporting Information 4

Plant families exhibit unique geographic trends in C_4_ richness and cover in Australia

Samantha E.M. Munroe*^1,2^, Francesca A. McInerney^3^, Greg R. Guerin^1,2^, Jake W. Andrae^3^, Nina Welti^4^, Stefan Caddy-Retalic^1,5^, Rachel Atkins^3^, & Ben Sparrow^1,2^

^1^ School of Biological Sciences, The University of Adelaide, Adelaide, South Australia 5005, Australia

^2^ Terrestrial Ecosystem Research Network (TERN), University of Adelaide, Adelaide, South Australia 5005, Australia

^3^ School of Physical Sciences and the Sprigg Geobiology Centre, The University of Adelaide, Adelaide, South Australia 5005, Australia

^4^ CSIRO Agriculture and Food, Urrbrae, South Australia 5064, Australia

^5^ School of Life and Environmental Sciences, University of Sydney, Sydney NSW 2006 Australia

*corresponding author: [samantha.munroe@adelaide.edu.au](mailto:samantha.munroe@adelaide.edu.au)

**S4: Binomial logistic regression model results of proportional C_4_ cover and richness analysis, and results of leave-one-out comparisons of the best fit models (i.e. “full model”) to predict proportional C_4_ cover and richness.** Predictor variables are mean annual temperature (MAT), mean annual precipitation (MAP), mean January minimum temperature (Jan. Min), mean January maximum temperature (Jan. Min), January precipitation (Jan. Precip), mean annual aridity index (Aridity), season water availability (SWA), mean annual proportion of C_4_ favoured months (Collatz) soil sand and clay content (%, sand, clay), pH, and available water capacity (AWC).

**C_4_ Poaceae Regression Analysis**

**S4 Table 1:** Binomial logistic regression model results of proportional C_4_ Poaceae cover analysis.

| **Variable** | **Estimate** | **Std Error** | **t value** | **p-value** |
| --- | --- | --- | --- | --- |
| **Intercept** | -20.60 | 3.41 | -6.05 | <0.001 |
| **Jan. Max** | 0.68 | 0.11 | 6.21 | <0.001 |
| **Jan. Precip** | 0.02 | 0.01 | 2.33 | 0.019 |
| **Tree Cover** | -0.03 | 0.02 | -1.38 | 0.162 |
| **AWC** | -0.14 | 0.14 | -1.01 | 0.315 |
| **Spatial autocovariate** | 2.36 | 0.51 | 4.60 | <0.001 |

**S4 Table 2:** Leave one out comparisons of the best fit model (i.e. “full model”) to predict proportional C_4_ Poaceae cover. The “Predictor Left Out” was removed from the full model and the QAIC was recalculated for the more simplified model.

| **Predictor Left Out** | **QAIC** | **∆QAIC** | **Weight** |
| --- | --- | --- | --- |
| **Jan. Max** | 621.88 | 144.4 | <0.001 |
| **Jan. Precip** | 495.53 | 18.06 | <0.001 |
| **Tree Cover** | 480.49 | 3.022 | 0.104 |
| **AWC** | 478.31 | 0.84 | 0.309 |
| **Full model** | 477.4 |  | 0.472 |

**S4 Table 3:** Binomial logistic regression model results of proportional C_4_ Poaceae richness analysis.

| **Variable** | **Estimate** | **Std Error** | **t value** | **p-value** |
| --- | --- | --- | --- | --- |
| **Intercept** | -14.74 | 2.24 | -6.56 | <0.001 |
| **Jan. Max** | 0.47 | 0.07 | 6.78 | <0.001 |
| **Jan. Precip** | 0.02 | 0.004 | 3.10 | 0.002 |
| **Tree Cover** | -0.02 | 0.01 | -1.66 | 0.096 |
| **Sand** | -0.03 | 0.01 | -2.55 | 0.01 |
| **Spatial autocovariate** | 3.44 | 0.42 | 8.10 | <0.001 |

**S4 Table 4**: Leave one out comparisons of the best fit model (i.e. “full model”) to predict proportional C_4_ Poaceae richness. The “Predictor Left Out” was removed from the full model and the QAIC was recalculated for the more simplified model.

| **Predictor Left Out** | **QAIC** | **∆QAIC** | **Weight** |
| --- | --- | --- | --- |
| **Jan. Max** | 563.79 | 82.54 | <0.001 |
| **Jan. Precip** | 501.03 | 19.78 | <0.001 |
| **Tree Cover** | 483.78 | 2.53 | 0.29 |
| **sand** | 491.30 | 10.05 | 0.01 |
| **Full model** | 481.25 | 0 | 0.77 |

**C_4_ Cyperaceae Regression Analysis**

**S4 Table 5**: Binomial logistic regression model results of proportional C_4_ Cyperaceae cover analysis.

| **Variable** | **Estimate** | **Std Error** | **t value** | **p-value** |
| --- | --- | --- | --- | --- |
| **Intercept** | -42.38 | 10.26 | -4.12 | <0.001 |
| **Jan. Min** | 1.15 | 0.19 | 5.96 | <0.001 |
| **Jan. Precip** | 0.02 | 0.01 | 3.01 | 0.003 |
| **Tree Cover** | -0.14 | 0.03 | -5.14 | <0.001 |
| **pH** | 3.11 | 1.21 | 2.57 | 0.011 |

**S4 Table 6**: Leave one out comparisons of the best fit model (i.e. “full model”) to predict proportional C_4_ Cyperaceae cover. The “Predictor Left Out” was removed from the full model and the QAIC was recalculated for the more simplified model.

| **Predictor Left Out** | **QAIC** | **∆QAIC** | **Weight** |
| --- | --- | --- | --- |
| **Jan. Min** | 722.25 | 563.87 | <0.001 |
| **Jan. Precip** | 183.60 | 25.232 | <0.001 |
| **Tree Cover** | 251.39 | 93.01 | <0.001 |
| **pH** | 185.98 | 27.60 | <0.001 |
| **Full model** | 158.37 | 0 | 0.99 |

**S4 Table 7**: Binomial logistic regression model results of proportional C_4_ Cyperaceae richness analysis.

| **Variable** | **Estimate** | **Std Error** | **t value** | **p-value** |
| --- | --- | --- | --- | --- |
| **Intercept** | -23.14 | 4.75 | -4.87 | <0.001 |
| **Collatz** | 11.65 | 1.45 | 7.99 | <0.001 |
| **Tree Cover** | 2.74 | 0.68 | 4.03 | <0.001 |
| **pH** | -0.09 | 0.01 | -6.33 | 0.011 |

**S4 Table 8:** Leave one out comparisons of the best fit model (i.e. “full model”) to predict proportional C_4_ Cyperaceae richness. The “Predictor Left Out” was removed from the full model and the QAIC was recalculated for the more simplified model.

| **Predictor Left Out** | **QAIC** | **∆QAIC** | **Weight** |
| --- | --- | --- | --- |
| **Collatz** | 628.01 | 469.16 | 0.013 |
| **Tree Cover** | 218.25 | 59.40 | <0.001 |
| **pH** | 195.34 | 36.49 | <0.001 |
| **Full model** | 158.84 | 0 | 1 |

**C_4_ Chenopodiaceae Regression Analysis**

**S4 Table 9**: Binomial logistic regression model results of proportional C_4_ Chenopodiaceae cover analysis.

| **Variable** | **Estimate** | **Std Error** | **t value** | **p-value** |
| --- | --- | --- | --- | --- |
| **Intercept** | 7.58 | 2.50 | 3.03 | 0.0026 |
| **Jan Max** | -0.05 | 0.03 | -1.65 | 0.099 |
| **Tree Cover** | -0.05 | 0.02 | -3.21 | 0.0014 |
| **pH** | -0.63 | 0.20 | -3.10 | 0.0021 |
| **Sand** | -0.04 | 0.01 | -3.61 | 0.0003 |
| **Spatial Autocovariate** | 1.095 | 0.33 | 3.28 | 0.0011 |

**S4 Table 10:** Leave one out comparisons of the best fit model (i.e. “full model”) to predict proportional C_4_ Chenopodiaceae cover. The “Predictor Left Out” was removed from the full model and the QAIC was recalculated for the more simplified model.

| **Predictor Left Out** | **QAIC** | **∆QAIC** | **Weight** |
| --- | --- | --- | --- |
| **Jan Max** | 311.58 | 0.48 | 0.400 |
| **Tree Cover** | 322.23 | 11.13 | 0.002 |
| **pH** | 318.32 | 7.22 | 0.012 |
| **Sand** | 321.50 | 10.40 | 0.003 |
| **Full Model** | 311.1 | 0 | 0.516 |

**S4 Table 11**: Binomial logistic regression model results of proportional C_4_ Chenopodiaceae richness analysis.

| **Variable** | **Estimate** | **Std Error** | **t value** | **p-value** |
| --- | --- | --- | --- | --- |
| **Intercept** | 1.18 | 0.486 | 2.44 | 0.0152 |
| **Jan. Precip** | -0.004 | 0.004 | -1.01 | 0.3120 |
| **Sand** | -0.04 | 0.007 | -6.19 | <0.001 |
| **Spatial Autocovariate** | 1.26 | 0.202 | 6.23 | <0.001 |

**S4 Table 12**: Leave one out comparisons of the best fit model (i.e. “full model”) to predict proportional C_4_ Chenopodiaceae richness. The “Predictor Left Out” was removed from the full model and the QAIC was recalculated for the more simplified model.

| **Predictor Left Out** | **QAIC** | **∆QAIC** | **Weight** |
| --- | --- | --- | --- |
| **Jan. Precip** | 487.00 | 2.42 | 0.020 |
| **Sand** | 508.96 | 24.39 | <0.001 |
| **Full Model** | 484.57 | 0 | 0.070 |

**C_4_ Euphorbiaceae Regression Analysis**

**S4 Table 13:** Binomial logistic regression model results of proportional C_4_ Euphorbiaceae cover analysis.

| **Variable** | **Estimate** | **Std Error** | **t value** | **p-value** |
| --- | --- | --- | --- | --- |
| **Intercept** | 38.05 | 394.69 | 0.096 | 0.923 |
| **AWC** | -0.85 | 15.41 | -0.056 | 0.956 |
| **Aridity** | -31.09 | 233.19 | -0.133 | 0.894 |
| **Jan. Max** | -0.79 | 10.19 | -0.078 | 0.938 |
| **Sand** | -0.065 | 1.85 | -0.035 | 0.972 |
| **SWA** | 19.66 | 204.63 | 0.096 | 0.924 |

**S4 Table 14:** Leave one out comparisons of the best fit model (i.e. “full model”) to predict proportional C_4_ Euphorbiaceae cover. The “Predictor Left Out” was removed from the full model and QAIC was recalculated for the more simplified model.

| **Predictor Left Out** | **QAIC** | **∆QAIC** | **Weight** |
| --- | --- | --- | --- |
| **AWC** | 120.61 | 6.417 | 0.02 |
| **Aridity** | 191.24 | 77.04 | <0.001 |
| **Jan. Max** | 133.89 | 19.69 | <0.001 |
| **Sand** | 115.18 | 0.98 | 0.36 |
| **SWA** | 141.19 | 26.99 | <0.001 |
| **Full Model** | 114.19 | 0 | 0.60 |

**S4 Table 15:** Binomial logistic regression model results of proportional C_4_ Euphorbiaceae richness analysis.

| **Variable** | **Estimate** | **Std Error** | **t value** | **p-value** |
| --- | --- | --- | --- | --- |
| **Intercept** | 12.33 | 147.90 | 0.083 | 0.934 |
| **AWC** | -0.97 | 13.34 | -0.073 | 0.942 |
| MAP | -0.018 | 0.14 | -0.123 | 0.903 |
| **SWA** | 9.58 | 141.35 | 0.068 | 0.946 |

**S4 Table 16:** Leave one out comparisons of the best fit model (i.e. “full model”) to predict proportional C_4_ Euphorbiaceae richness. The “Predictor Left Out” was removed from the full model and QAIC was recalculated for the more simplified model.

| **Predictor Left Out** | **QAIC** | **∆QAIC** | **Weight** |
| --- | --- | --- | --- |
| **AWC** | 120.64 | 8.81 | 0.011 |
| **MAP** | 169.63 | 57.80 | <0.001 |
| **SWA** | 120.71 | 8.88 | 0.011 |
| **Full Model** | 111.82 | 0 | 0.96 |
