# Supplementary material for: Plant families exhibit unique geographic trends in C4 richness and cover in Australia
Source: PLoS One. 2022 Aug 22;17(8):e0271603. doi: 10.1371/journal.pone.0271603 (PMC9394836; doi:10.1371/journal.pone.0271603)
Supplement: S1 File — (DOCX) [file pone.0271603.s004.docx]

Supporting Information 3

Maps of proportional C_4_ cover and richness at TERN Monitoring plots for additional 10 families in which C_4_ species were identified

Plant families exhibit unique geographic trends in C_4_ richness and cover in Australia

Samantha E.M. Munroe^1,2^, Francesca A. McInerney^3^, Greg R. Guerin^1,2^, Jake W. Andrae^3^, Nina Welti^4^, Stefan Caddy-Retalic^1,5^, Rachel Atkins^3^, & Ben Sparrow^1,2^

^1^ School of Biological Sciences, The University of Adelaide, Adelaide, South Australia 5005, Australia

^2^ Terrestrial Ecosystem Research Network (TERN), University of Adelaide, Adelaide, South Australia 5005, Australia

^3^ School of Physical Sciences and the Sprigg Geobiology Centre, The University of Adelaide, Adelaide, South Australia 5005, Australia

^4^ CSIRO Agriculture and Food, Urrbrae, South Australia 5064, Australia

^5^ School of Life and Environmental Sciences, University of Sydney, Sydney NSW 2006 Australia


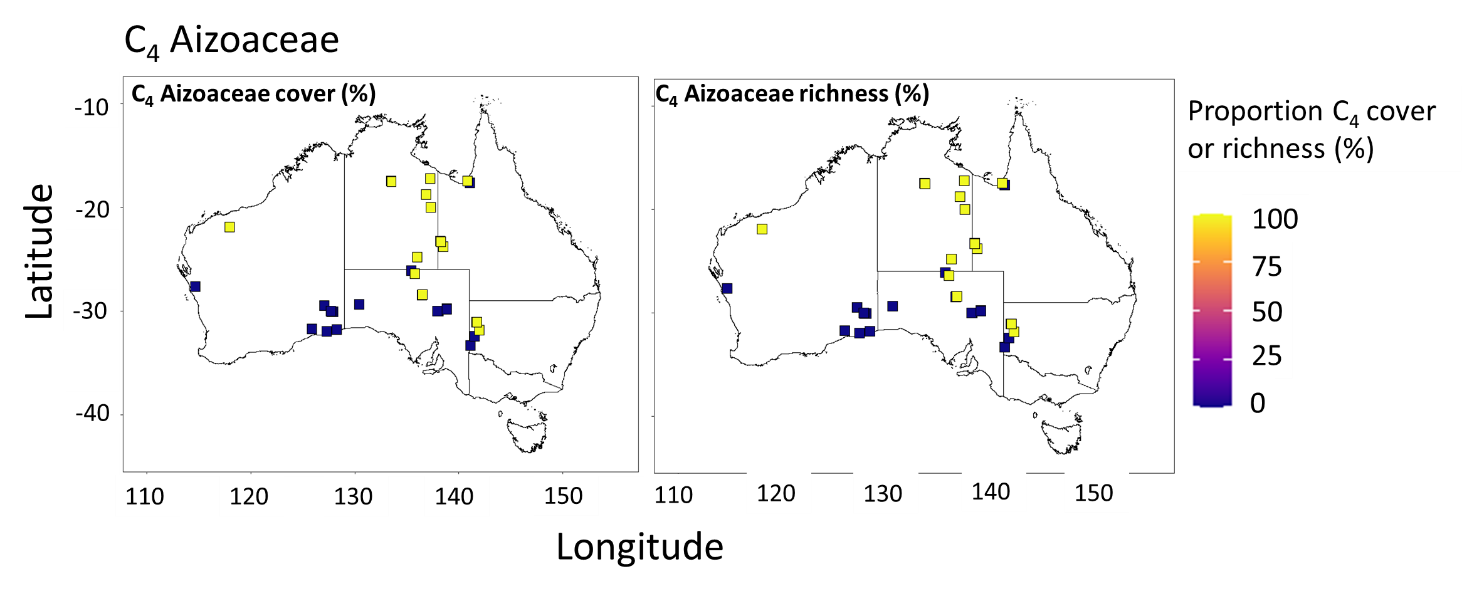


**Supporting Information 3 Figure 1: Proportional (%) Aizoaceae C_4_ cover and richness at TERN Monitoring plots**


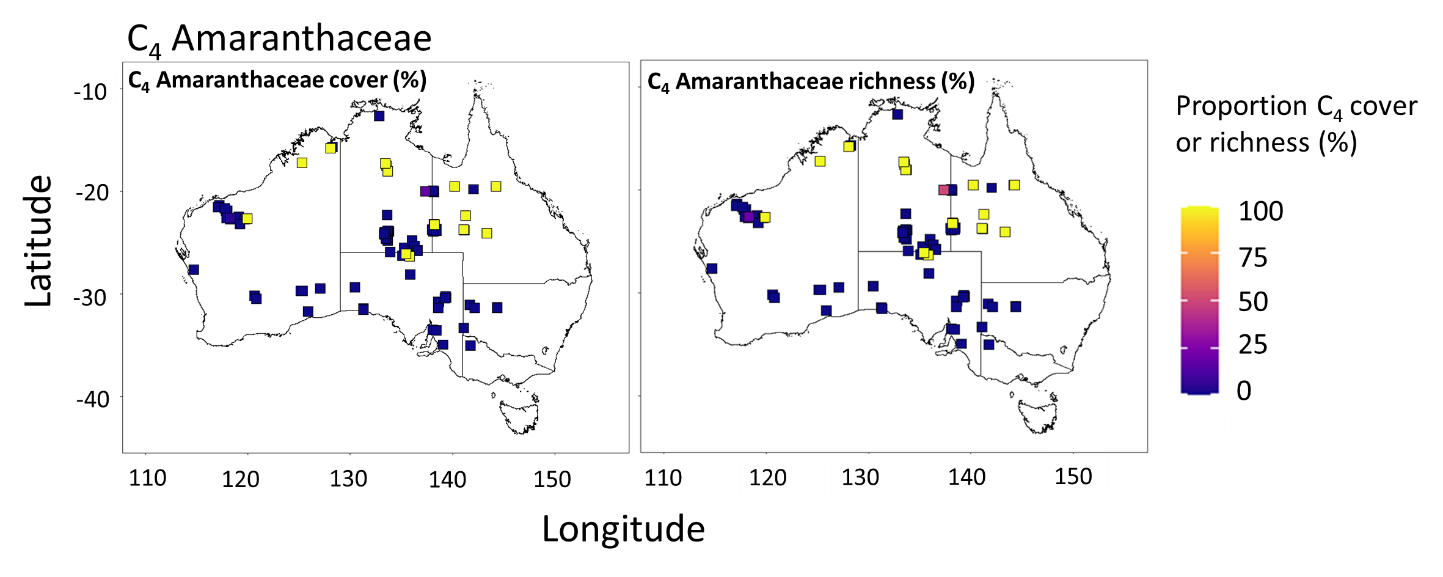


**Supporting Information 3 Figure 2: Proportional (%) Amaranthaceae C_4_ cover and richness at TERN Monitoring plots**


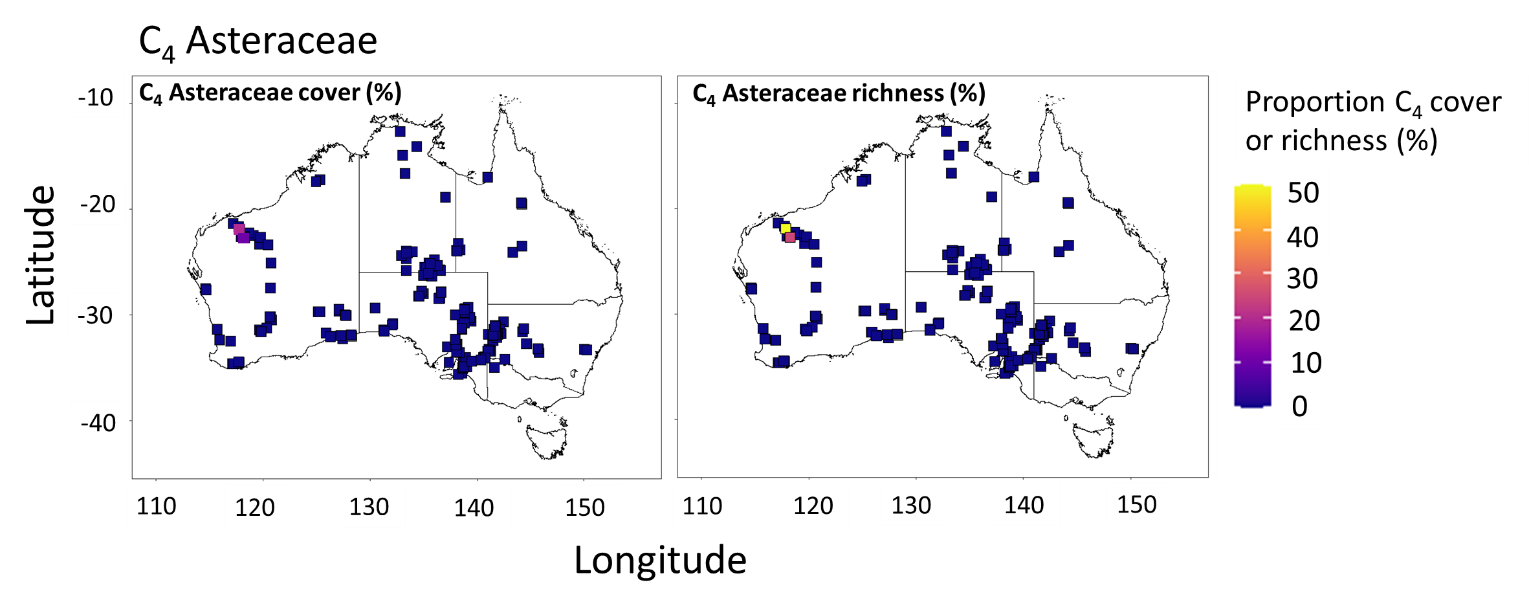


**Supporting Information 3 Figure 3: Proportional (%) Asteraceae C_4_ cover and richness at TERN Monitoring plots**


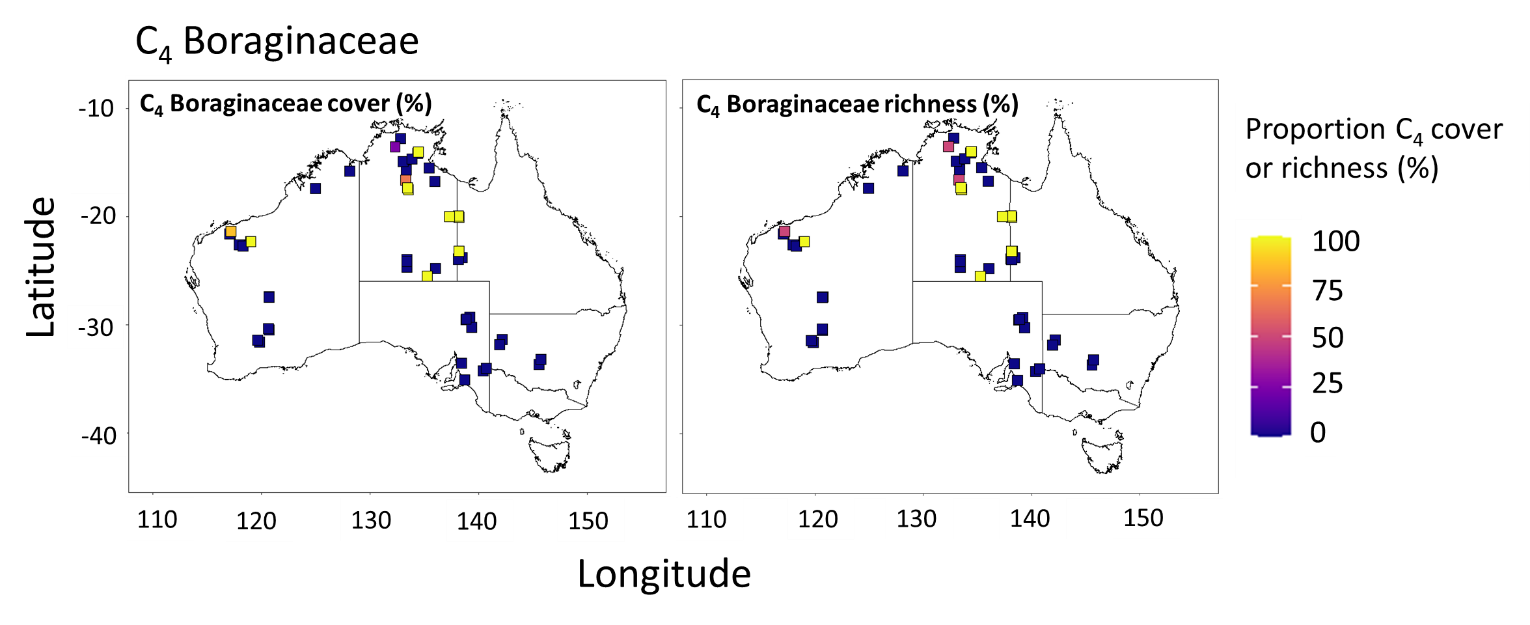


**Supporting Information 3 Figure 4: Proportional (%) Boraginaceae C_4_ cover and richness at TERN Monitoring plots**


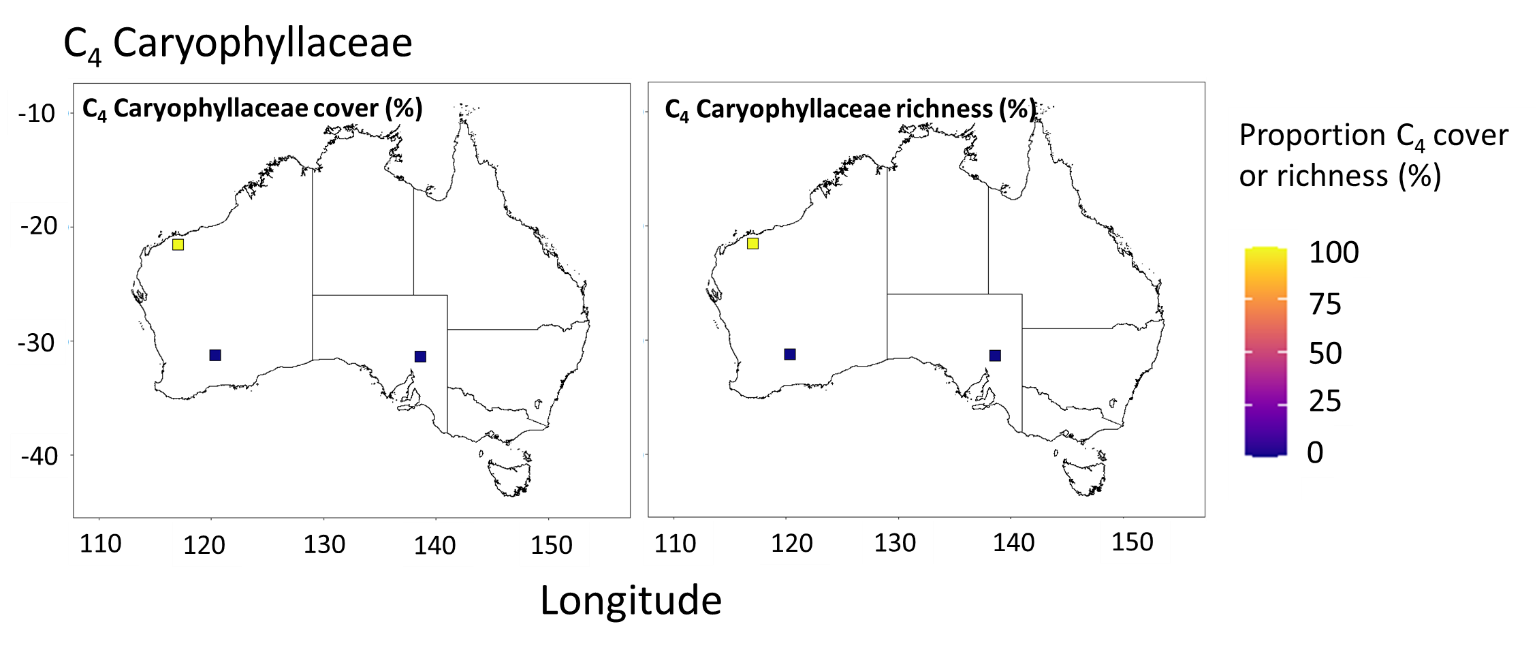


**Supporting Information 3 Figure 5: Proportional (%) Caryophyllaceae C_4_ cover and richness at TERN Monitoring plots**


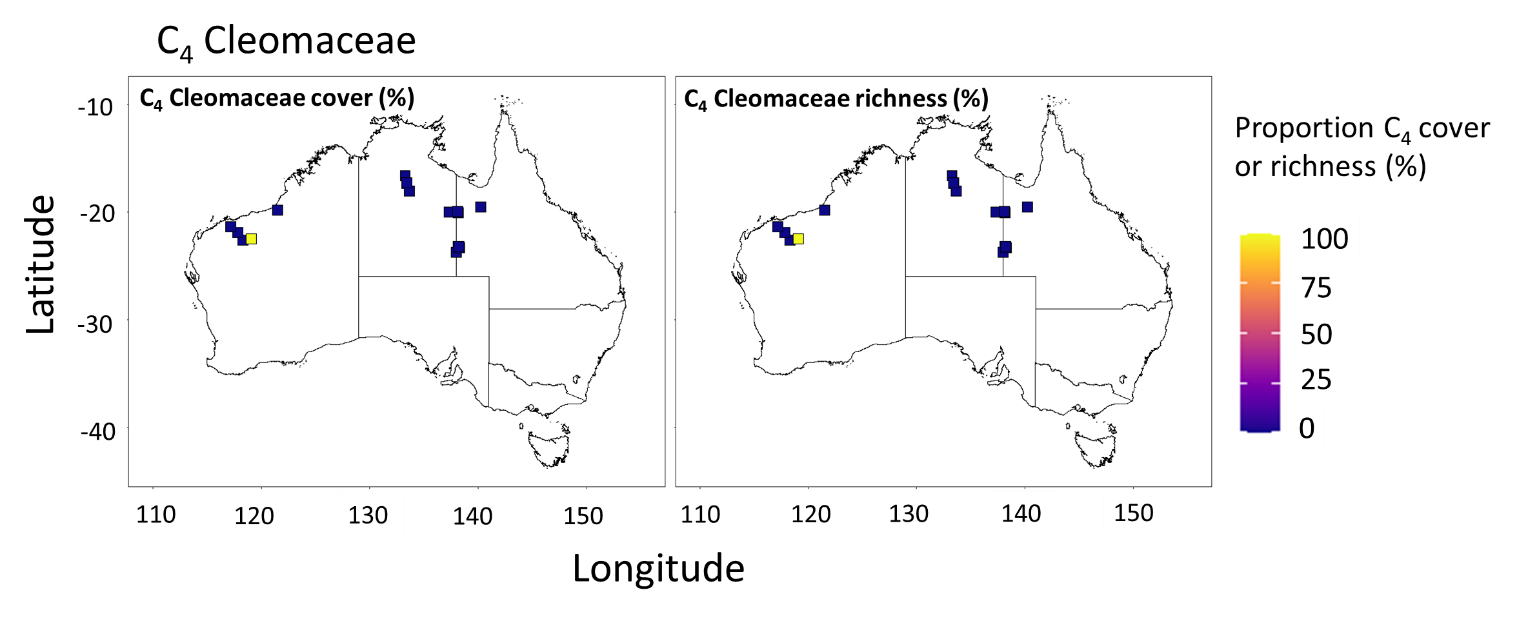


**Supporting Information 3 Figure 6: Proportional (%) Cleomaceae C_4_ cover and richness at TERN Monitoring plots**


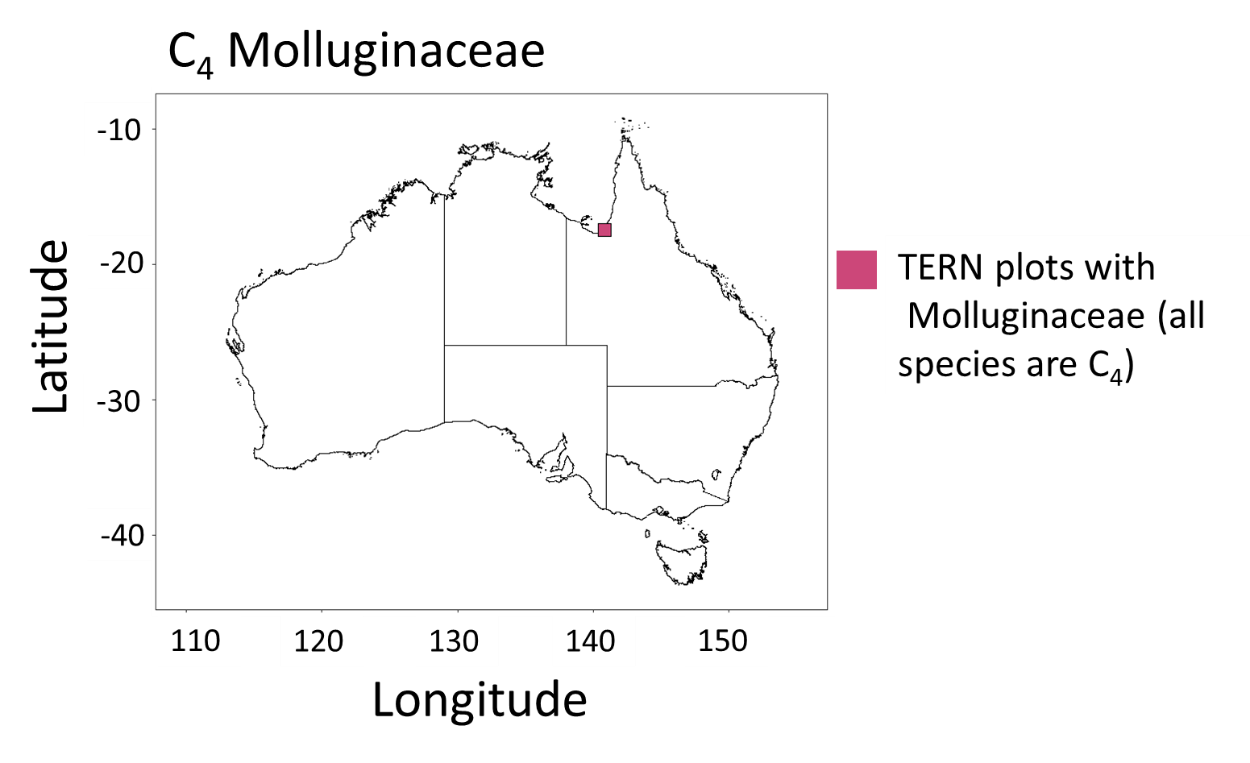


**Supporting Information 3 Figure 7: TERN Monitoring plots where Molluginaceae was identified. All Molluginaceae have C_4_ photosynthesis**


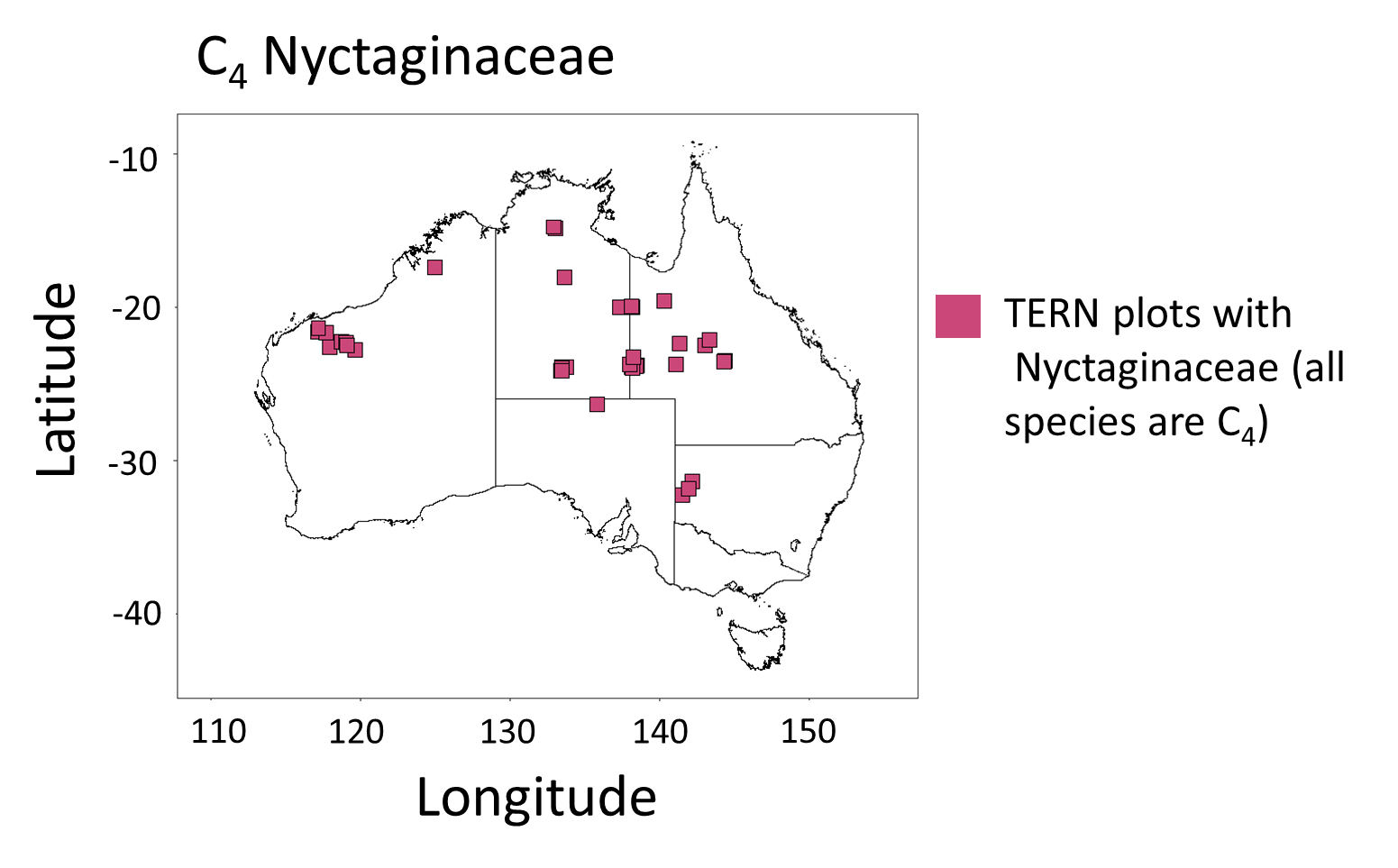


**Supporting Information 3 Figure 8: TERN Monitoring plots where Nyctaginaceae was identified. All Nyctaginaceae have C_4_ photosynthesis**


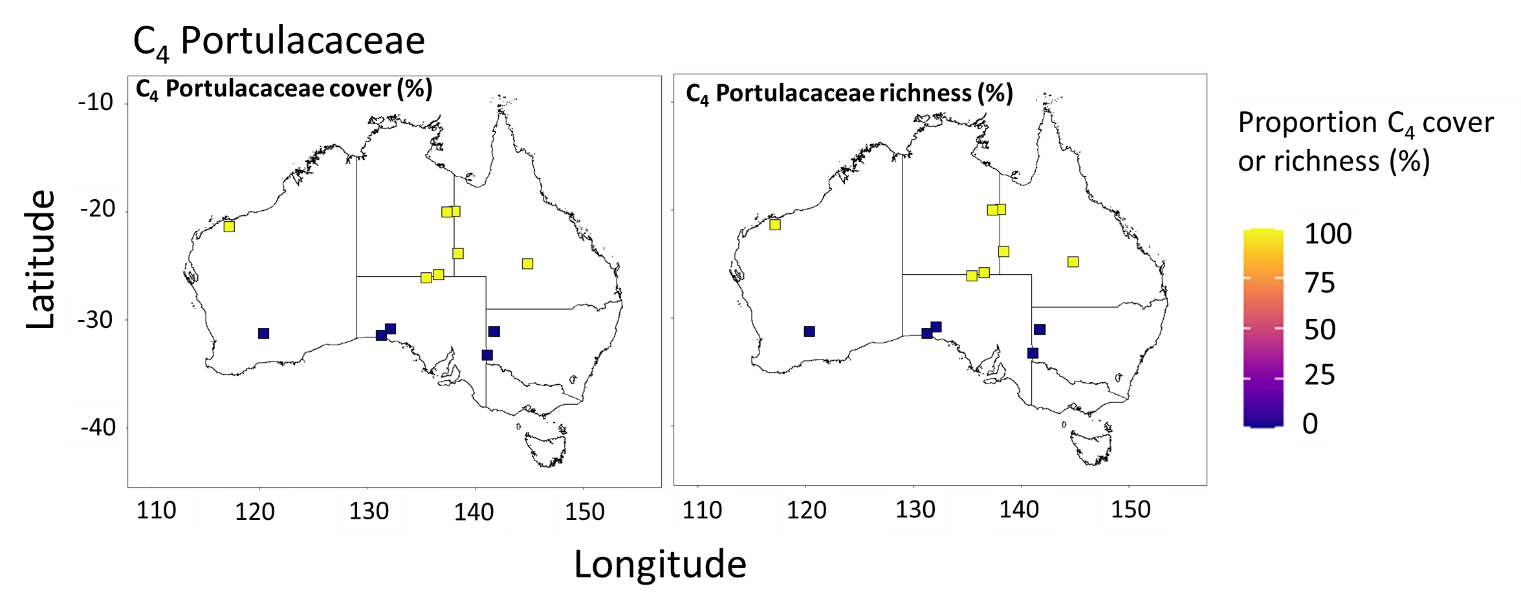


**Supporting Information 3 Figure 9: Proportional (%) Portulacaceae C_4_ cover and richness at TERN Monitoring plots**


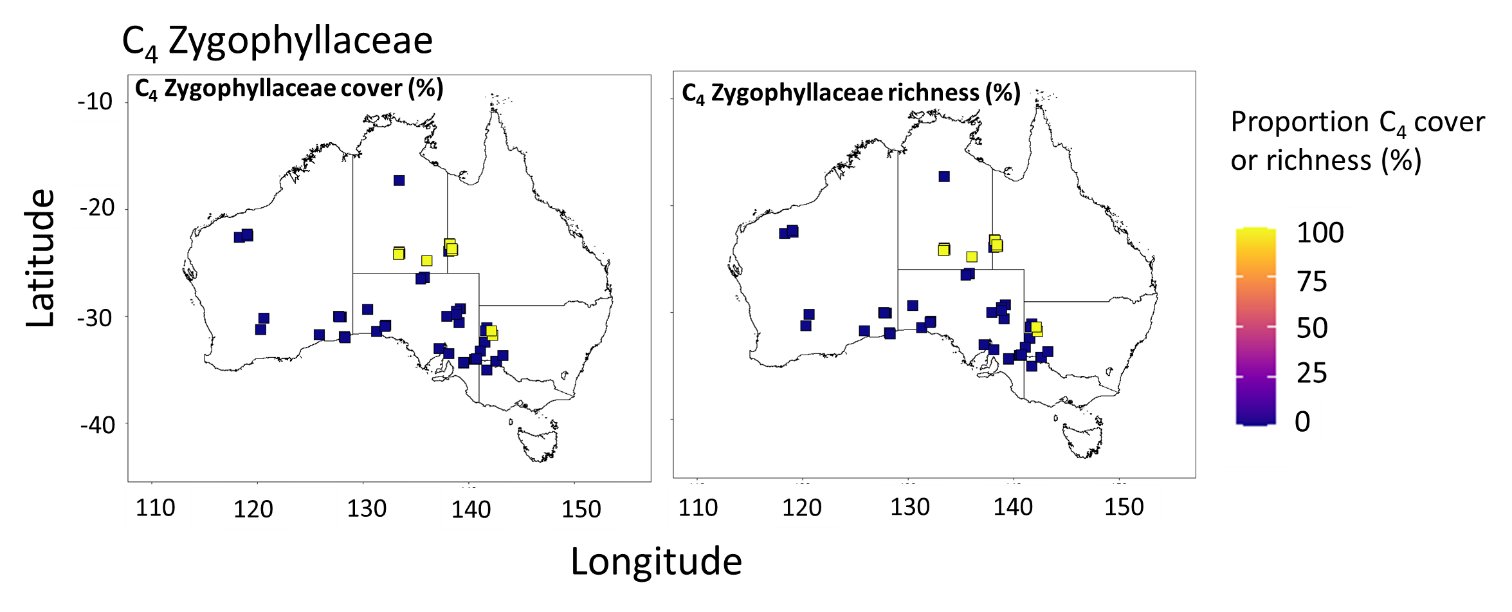


**Supporting Information 3 Figure 10: Proportional (%) Zygophyllaceae C_4_ cover and richness at TERN Monitoring plots**
