# Supplementary material for: Plant families exhibit unique geographic trends in C4 richness and cover in Australia
Source: PLoS One. 2022 Aug 22;17(8):e0271603. doi: 10.1371/journal.pone.0271603 (PMC9394836; doi:10.1371/journal.pone.0271603)
Supplement: S2 File — (DOCX) [file pone.0271603.s005.docx]

Supporting Information 5

Plant families exhibit unique geographic trends in C_4_ richness and cover in Australia

Samantha E.M. Munroe*^1,2^, Francesca A. McInerney^3^, Greg R. Guerin^1,2^, Jake W. Andrae^3^, Nina Welti^4^, Stefan Caddy-Retalic1,5, Rachel Atkins^3^, & Ben Sparrow^1,2^

^1^ School of Biological Sciences, The University of Adelaide, Adelaide, South Australia 5005, Australia

^2^ Terrestrial Ecosystem Research Network (TERN), University of Adelaide, Adelaide, South Australia 5005, Australia

^3^ School of Physical Sciences and the Sprigg Geobiology Centre, The University of Adelaide, Adelaide, South Australia 5005, Australia

^4^ CSIRO Agriculture and Food, Urrbrae, South Australia 5064, Australia

^5^ School of Life and Environmental Sciences, University of Sydney, Sydney NSW 2006 Australia

*corresponding author: [samantha.munroe@adelaide.edu.au](mailto:samantha.munroe@adelaide.edu.au)

**
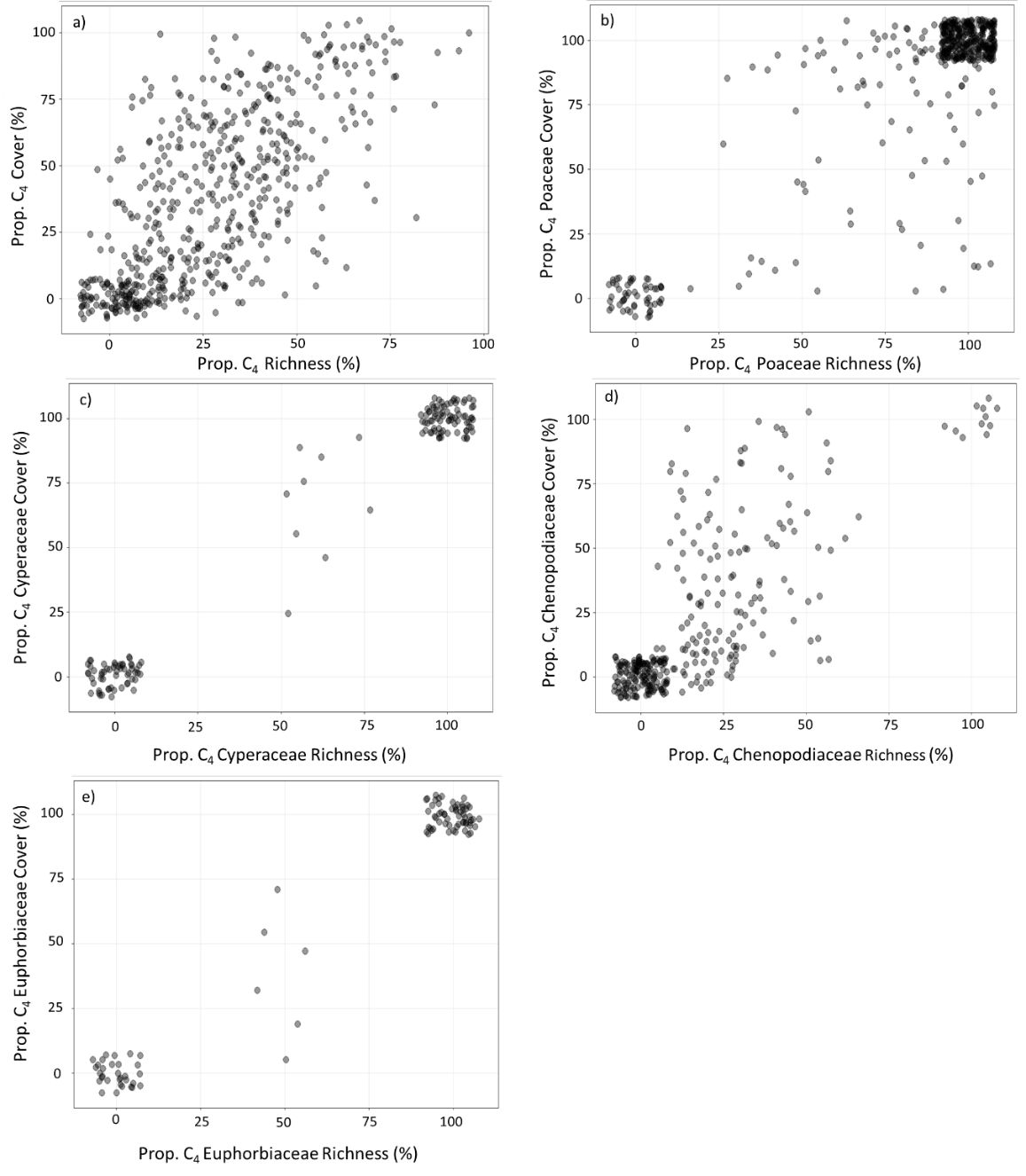
**

**S5.** **Proportional C_4_ richness versus cover for a) all species, b) Poaceae, c) Cyperaceae, d) Chenopodiaceae and e) Euphorbiaceae.** Points do not indicate exact values. Points have been displaced to clarify the number of 0 and 1 values.
